# Genome-wide identification and transcription profiling of safflower (*Carthamus tinctorius* L.) HD-ZIP gene family under water deficit

**DOI:** 10.1186/s12864-025-12060-4

**Published:** 2025-09-29

**Authors:** Fahime Sabzeali, Asadollah Ahmadikhah, Naser Farrokhi, Reza Haghi

**Affiliations:** 1 Department of Cellular & Molecular Biology, Faculty of Life Sciences and Biotechnology, Shahid Beheshti University, Tehran, Iran; 2 Department of Gene Bank, Leibniz Institute of Plant Genetics and Crop Plant Research, Seeland, OT Gatersleben Germany

**Keywords:** Abiotic stress, Homeodomain leucine zipper, Promoter analysis, RNA-seq, Safflower, transcription factor

## Abstract

Safflower (*Carthamus tinctorius* L.) is a multipurpose oilseed crop with high nutritional value and natural tolerance to stressful conditions including drought. The homeodomain leucine zippers (HD-ZIPs) are a class of highly conserved DNA-binding domains in plant transcription factors (TFs) which involve in different biological processes including growth, development, signal transduction and response to abiotic stresses. To date, the HD-ZIP transcription factors in safflower weren’t characterized in a genome-wide scale and their role in response to drought stress is unknown. In this study, 46 HD-ZIP coding genes were identified across the safflower genome, and gene structures, protein characteristics, conserved motifs, duplication and syntenic patterns, and protein-protein interactions were explored. The TFs were classified into four main subfamilies (I to IV) based on phylogenetic analyses. Segmental duplication has played a prominent role in the expansion of the HD-ZIP TFs. Promoter analysis of the genes highlighted the prevalence of ABRE, MYB, MYC, ARE and STRE *cis*-elements. Most of safflower HD-ZIPs interact with each other or with other TFs and functional proteins involved in organogenesis and crucial biological processes. The expression profile of safflower HD-ZIPs was explored in seedlings under drought stress using RNA-seq assay. Four genes from HD-ZIP I subfamily including *CtHDZIP23* (*HB-7*), *CtHDZIP7* (*HB-7*), *CtHDZIP26* (*HAT5*) and *CtHDZIP3* (*HB-40*) were significantly upregulated in response to drought stress, and two genes in the subfamily (*CtHDZIP9*, *CtHDZIP41*; *HB13*) were significantly downregulated. However, in HD-ZIP II subfamily only *CtHDZIP37* (*HAT22*) was significantly upregulated. These results were confirmed using real-time qRT-PCR. Our findings highlight the members of the HD-ZIP gene family in response-regulation of drought stress, and will significantly help in understanding the function of the HD-ZIP TFs in response to abiotic stresses in safflower.

**Supplementary Information:**

The online version contains supplementary material available at 10.1186/s12864-025-12060-4.

## Introduction

With progressive climate change, drought has become a prominent stress that limits plant growth and development, leading to considerable economic damages [1]. One way to counteract its impact and ensure the world’s food security would be breeding for drought-tolerant crops [2]. Plants have evolved complex regulatory mechanisms to cope with drought stress, which is a major abiotic factor limiting crop productivity. The key regulatory mechanisms include signal perception and transduction [3, 4], hormonal regulation [5], transcriptional networks [6], physiological and biochemical adaptations [7], and molecular and genetic regulation [8]. Safflower (*Carthamus tinctorius* L.) is an important oilseed crop that is naturally adapted to arid and semi-arid environments, making it a valuable model for studying drought tolerance [9]. Despite its inherent tolerance, drought stress still significantly reduces safflower’s growth, yield, oil content, and quality [10, 11]. For example, drought stress in safflower during the vegetative growth, flowering and seed-filling stages surprisingly reduces the yield [12]. In comparison to other oilseeds, safflower is relatively drought-tolerant. Meanwhile, the molecular mechanisms underlying safflower’s better adaptation to drought stress are mostly unknown. Understanding the regulatory mechanisms enables breeders to identify and select for drought-tolerant genotypes, using physiological indices and molecular markers [13]. Marker-assisted selection and genomic studies can accelerate the development of resilient safflower varieties suited for water-limited regions [9]. With increasing water scarcity and climate change, crops like safflower that can maintain productivity under drought are critical for sustainable agriculture and food security [10]. Insights from safflower can be translated to improve drought tolerance in other crops. Compared to major crops, the molecular basis of drought tolerance in safflower is less understood, highlighting the need for more research to unlock its full potential.

**Fig. 1:**
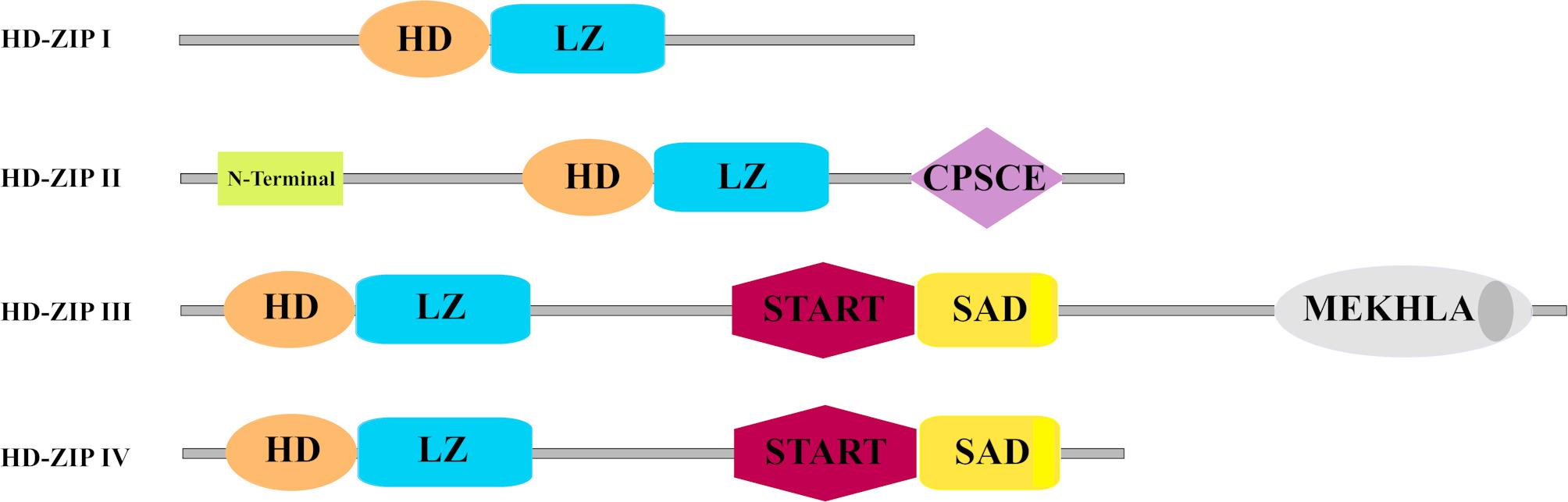
Protein structure of different sub-families of plant HD-ZIPs. HD, homeodomain; LZ, leucine-zipper; N-Term, N terminus consensus sequence; CPSCE, Cys-Pro-Ser-Cys-Glu conserved domain; START, steroidogenic acute regulatory protein; MEKHLA, Met-Glu-Lys-His-Leu-Ala conserved domain; SAD, START-associated domain [31]

Comprehension of gene regulatory systems in plants is indispensable to improving tolerance to abiotic stresses. The homeodomain leucine zipper (HD-ZIP) regulates the response to abiotic stresses, signal transduction, growth and development [14, 15]. For instance, HD-ZIP family members such as *HaHB4* of sunflower [16], *AtHB7* and *AtHB12* [17, 18], and *HAT1* [19] in Arabidopsis, and *TaHDZ5-6 A* in bread wheat [20] have shown to be up-regulated under drought stress. All members of the HD-ZIP family consist of a homeodomain (HD) and leucine zipper (LZ) motif. HD with 61 amino acid residues creates a helical structure that recognizes specific promoter regions [21, 22], while LZ is an α-helix with seven amino acid residues that provide dimerization of HD-ZIP proteins [23]. DNA binding domain is located at the N-terminus of HD and the C-terminus of LZ [24]. Depending on the sequence motifs and number of exons/introns, HD-ZIP TFs are arranged into four subfamilies I, II, III, and IV in *Arabidopsis thaliana* (Supplementary Table S1). In the HD-ZIP I subfamily there are two main motifs HD and LZ (Fig. 1). In the HD-ZIPI II subfamily, in addition to the HD and LZ main domains, CPSCE motifs with Cys, Pro, Ser, Cys, and Glu amino acids are found at the N-terminus and play an important role in response to abiotic stresses [25–28]. The motifs of SAD and START are found in HD-ZIP III and IV subfamilies [29–36]. The START domain involves in ABA-response [29]. The MEKHLA motif in the C-terminus is an exclusive member of HD-ZIP III [30].

The adverse environmental conditions, especially drought stress, affect gene expression of the HD-ZIP I subfamily [37–43]. The *MtHB1* gene in *Medicago truncatula* from HD-ZIP I class adjusts root structure in environmental stress by suppressing LBD1 (lab binding domain 1) [44]. The *OsTF1* (from HD-ZIP I subfamily) overexpression in transgenic rice led to enhanced lignin biosynthesis, stomatal closure, and induction of responsive genes under drought stress [45]. The upregulation of *NaHD20* gene in tobacco leaves under drought stress leads to the accumulation of abscisic acid and the induction of genes responsive to dehydration stress [46]. In the present research, we report for the first time, the characterization of HD-ZIP family and their gene expression patterns in safflower seedlings experiencing drought stress. Fourty six safflower HD-ZIPs were classified in to four subfamilies (I to IV) and significant synteny patterns were detected among HD-ZIPs of safflower and other plant species. Five pairs of duplicated genes were identified in safflower genome, most of which were emerged due to segmental (genome-wide) expansion. Most of safflower HD-ZIP proteins show interaction with each other or with other TFs and functional proteins that are involved in organogenesis and vital biological processes. Amongst members of different HD-ZIP subfamilies, only members of subfamily I (*n* = 7) and II (*n* = 1) responded to drought stress at seedling stage.according to RNAseq assay. The findings can be utilized in breeding and improvement programs for safflower cultivars towards higher drought tolerance. Our findings can lead to the identification of a subset of the HD-ZIP genes to regulate the genes involved in drought tolerance.

## Materials and methods

### Safflower HD-ZIPs identification

The protein sequences of safflower (*Carthamus tinctorius* L.) were obtained from safflower database (https://safflower.scuec.edu.cn/). Three species of the Asteraceae family, including sunflower (*Helianthus genus* L.), lettuce (*Lactuca sativa* L.), and sesame (*Sesamum indicum* L.), along with *A. thaliana* (from Brassicaceae family as outgroup) were selected for the study. The HD-ZIP protein sequences of sunflower, sesame, and lettuce were downloaded from the https://plantregmap.gao-lab.org/ database and used in homology and phylogenetic analyses. HD-ZIP protein sequences of *A. thaliana* were downloaded from the Arabidopsis Information Resource (https://www.arabidopsis.org/). Multiple sequence alignment (MSA) among Arabidopsis HD-ZIP proteins was conducted using Mega v.11. The file containing MSA results was used to generate HMM profile at HMMER online software (https://www.ebi.ac.uk/Tools/hmmer/), and the builded HMM profile was used for identification of HD-ZIP proteins in safflower genome (with E-value < 10^−5^ for ocurrence of HD-ZIP homeobox domain) [47].

Classification of HD-ZIP protein families (IPR044830) was downloaded from the InterPro 97.0 database (https://www.ebi.ac.uk/interpro). The Hidden Markov Model (HMM) profile of the Homeobox (PF00046) and HD-ZIP domain (PF04618) was obtained from Pfam database (http://pfam.sanger.ac.uk) [48]. SMART (https://smart.embl.de/) [49] was used to confirm the HD-ZIP proteins, and then redundant sequences were removed from data set. Physiochemical features of HD-ZIP proteins such as molecular weight (MW) and isoelectric point (PI) were obtained at https://web.expasy.org/ProtParam/. The subcellular localization of HD-ZIP proteins in safflower was predicted by WoLF PSORT (http://wolfpsort1.hgc.jp/form.html).

### Conserved motif analysis and determining the 3D models

The HD-ZIP proteins conserved motifs were characterized by MEME [50] at (http://meme-suite.org/tools/meme) with parameters adjusted as a maximum number of motifs to 50, the minimum and maximum width of the motifs ranging from 6 to 200. Main and secondary motifs, the number and role of each motif were used in group classification. Using the SMART database (http://smart.embl-heidelberg.de/) the structure of conserved domains of HD and LZ was determined.

To determine 3D models of HD-ZIP proteins, online tools at https://swissmodel.expasy.org/ were used. Gene ontology of HD-ZIP proteins was performed using g: Profiler website (16; https://biit.cs.ut.ee/gprofiler/).

### Analysis of phylogenetic relationships, synteny and gene duplication

Molecular Evolutionary Genetic Analysis (MEGA11) was used following ClustalW alignment to construct a phylogenetic tree based on the Neighbor-Joining and the best-fit model of Jones-Taylor-Thornton (JTT) + G + F with 1000 replicates in bootstrap analysis [51]. The iTOL v6 online tool (https://itol.embl.de/) was used to reconstruct the final phylogenetic tree.

The protein sequences of HD-ZIPs for synteny analysis were downloaded from the online database https://plantregmap.gao. Synteny analysis was performed with Circoletto online tool following default parameters (https://bat.infspire.org/circoletto/) and TBtool v.2.083 software.

The locus relationship of homologous HD-ZIP genes and duplication events were checked by analyzing covariance relationship among homologous gene pairs using MCScanX [52]. To investigate selection pressure on tandem or segmentally duplicated genes, Ka (non-synonymous)/Ks (synonymous) ratios (Ka/Ks) were calculated between the pairs of homologous genes by TBtools v.2.083 and RStudio version 4.3.3 (seqinr package).

### Gene structure and genomic locations of HD-ZIP TFs

Gene Structure Display Server (http://gsds.gao-lab.org/) was applied to diagnose the position of exon and intron structure of genes based on GTF/GFF3 file annotations. To identify *Cis*-regulatory elements in promoters regions of HD-ZIP genes and their possible role in the rate and process of gene expression in creating tolerance to drought stress, 2000 bp upstream of the genes were analyzed at (https://bioinformatics.psb.ugent.be/webtools/plantcare/html/). To recognize the position and distribution of forty-seven HD-ZIP genes on twelve chromosomes of safflower, the MapChart232 software [53] was used.

### Gene ontology (GO) enrichment and constructing protein-protein interaction (PPI) network

The statistical analysis of gene ontology [16], with *p*-value < 0.05 and Kyoto Encyclopedia of Genes and Genomes (KEGG) pathways were performed using the g: Profiler tool (https://biit.cs.ut.ee/gprofiler/gost) to determine drought stress-affected biological processes. Gene ontologies including molecular function (MF), biological process (BP) and cellular component (CC) were determined in this analysis. The protein-protein interaction (PPI) network of safflower HD-ZIP genes from the perspective of *A. thaliana* homologous unigenes was constructed using STRING tool (https://string-db.org/).

### Drought treatment and RNA isolation

Safflower cv. Arak 2811 seeds were collected from the oilseed section of the Seed and Plant Improvement Institute (SPII) (Alborz, Iran). The seeds were surface-sterilized using sodium hypochlorite (1%) for 5 min and washed several times with dH_2_O. Uniformly sterilized seeds were planted in 800 g dark pots (with a diameter of 20 cm) with sterilized soil and free of weed seeds in the research greenhouse of the weed section of the National Plant Protection Research Institute at ambient temperature (‌25 ± 2‌‌°C) and dark/light regim of 16‌ h/8 h. In each pot, 10 healthy seeds were grown. The pots were regularly watered every three days. During leaf emergence and in the 5–8 leaf stage of safflower (38 days after planting), drought stress was applied by withholding water for 10 days under controlled conditions. After observation of twisting, wilting, and leaf rolling in safflower leaves (indication of drought stress), fresh leaves were collected and washed, snap-frozen in liquid nitrogen and stored at −80 °C until RNA extraction.

Total RNA was extracted from drought-treated and control plant leaves (from 5 plants in each replicate) using a QIAGEN kit^®^ (Kit Mini Plant) according to the manufacturer’s protocols. Briefly, 100 mg of plant tissue was used and macerated in an extraction buffer containing 1% β-mercaptoethanol. RNA quality and quantity were evaluated using 1% agarose gel and NanoDrop spectrophotometry (Thermo Fisher Scientific, Waltham, ‌MA, ‌USA). The RNA integrity number (RIN) was determined by microcapillary electrophoresis separation. An 18 S/28S ratio above 2, an OD 260/280 ratio greater than 1.8, and a RIN > 7 were the basic factors for selecting RNA for sequencing. According to the manufacturer’s protocol, cDNA synthesis was performed using AddScript kit^®^ (Daejeon, South Korea). RNA-seq libraries were prepared after cDNA synthesis using oligo dT method.

### RNA-seq profiling of differentially expressed HD-ZIP genes

RNA sequencing was accomplished with the Illumina Novaseq 6000 platform using Flow cell SP XP in round-trip (with 100× two cycles, using paired-end method) in two independent biological replicates, and short reads were saved in FASTQ format. Read length was 150 bp. Trimmomatic software was utilized for removing adapters and reads of inappropriate quality, and the quality of trimmed reads was scrutinized using FASTQC v0.11.9 [54]. The reference genome of safflower (safflower.genome.v.1) was downloaded from http://safflower.scuec.edu.cn. All the clean reads were mapped against the genome using HiSat2. Transcript counts were determined using StringTie software [55].

The counts of each sample were converted to count per million (CPM) [56, 57]. To remove any batch effect, CPM data was normalized in R software version 4.2.2 with the edgeR package [58]. DEGs were identified using the glmFit function in R version 4.2.2 with edgeR and limma packages [59]. To claim the significance of genes the |log2 fold change| ≥ 1 and *adjP* ≤ 0.05 were considered.

### Real-time qRT-PCR of HD-ZIPs

To validate the reliability results of RNA-seq, six genes from a list of top genes including four up- and two down-regulated DEGs and a housekeeping gene of safflower (*EF1*) [60] were chosen to be checked by real-time qRT-PCR in three replicates using gene-specific primer pairs (Supplementary Table S2). Real-time PCR was performed in BioRad CFX-96™ Real-time PCR and the REST 2009 software was used to calculate the relative expression of selected genes [61].

## Results and discussion

### Identification of HD-ZIP proteins in C. tinctorius

Using *A. thaliana* HD-ZIP HMM profile against protein sequences encoded by safflower genome resulted in preliminary identification of 50 safflower HD-ZIP genes. After removing redundant sequences, forty six HD-ZIP genes were predicted in safflower, homologous to 19 unigenes of HD-ZIPs in Arabidopsis (Supplementary Table S3). Then, the identified HD-ZIP proteins were examined with InterProScan (https://www.ebi.ac.uk/interpro/search) to determine HD and LZ domains, that confirmed these features in the protein sequences of safflower HD-ZIPs.

In total, 214 HD-ZIP protein sequences from five plant species including safflower, sunflower, lettuce, sesame and Arabidopsis consisted our data set for subsequent analyses. The members of HD-ZIP I, II, III and IV subfamilies in safflower, respectively, were enumerated 22, 12, 5, and 7 (Supplementary Table S3). This distribution of HD-ZIP members was almost similar to *A. thaliana* (I: *n* = 17, II: *n* = 9, III: *n* = 5 and IV: *n* = 16). Most genes belonged to HD-ZIP I subfamily and the least belonged to the HD-ZIP III subfamily. However, the numbers of HD-ZIP genes of *Lactuca sativa* in subfamilies I, II. III and IV were 12, 9, 4 and 5, respectively. In *Sesamum indicum*, 19, 13,13 and 20 genes were discovered, respectively, in subfamilies I, II, III, and IV. In *Helianthus anus*, 11, 3, 0 and 1 genes were identified, respectively, in subfamilies I, II.III and IV.

The homeodomain of HD-ZIP proteins (PF04618) in safflower and other plant species was similar. The number of amino acids in the proteins of safflower ranged from 152 to 848 aa. The *CtHDZIP20* (*Ct5T0237900.1*) had the lowest number of amino acids (*n* = 152 aa), and *CtHDZIP42* (*Ct11T0164800.1*) had the highest number of amino acids (*n* = 848 aa) (Table 1). The molecular weight ranged from 17,494.54 to 93,289.39 KDa, the theoretical PI ranged from 4.36 to 9.3, and the instability index ranged from 39.55 to 74.22. As expected, the cellular localization of most HD-ZIP genes according to their expected role in the regulation of gene expression was nuclear (85%) (Table 1).

**Table 1:** Genomic position, number of amino acids, molecular weight, isoelectric point (PI), instability index and subcellular localization of HD-ZIP proteins identified in *C. tinctorius*

CtHDZIP name	Gene ID	E-value of homeobox existence	Chromosome	Start position (bp)	End position (bp)		Number of amino acids	MW	PI	instability index	Subcellular localization
*CtHDZIP1*	*Ct1T0013500.1*	4.50E-16	1	1,635,756	1,641,720		306	34733.94	5.61	53.42	Nuclear
*CtHDZIP2*	*Ct1T0037200.1*	1.40E-15	1	5,072,879	5,077,774		290	32123.92	8.61	52.95	Nuclear
*CtHDZIP3*	*Ct1T0047300.2*	6.50E-17	1	7,031,741	7,035,062		207	23913.07	5.9	54.6	Nuclear
*CtHDZIP4*	*Ct1T0083900.1*	3.80E-17	1	15,387,184	15,389,014		308	34284.77	4.75	59.41	Cytoplasmic
*CtHDZIP5*	*Ct1T0166900.1*	5.10E-16	1	64,536,788	64,545,102		355	39483.26	9.00	74.22	Nuclear
*CtHDZIP6*	*Ct1T0229600.1*	5.00E-17	1	86,194,648	86,204,506		700	77210.23	5.58	46.99	Nuclear
*CtHDZIP7*	*Ct2T0031800.1*	6.00E-16	2	3,915,780	3,916,781		206	24240.79	5.83	71.46	Nuclear
*CtHDZIP8*	*Ct2T0122800.1*	1.70E-16	2	22,480,711	22,483,553		296	34708.34	4.79	63.46	Nuclear
*CtHDZIP9*	*Ct2T0125800.1*	1.40E-15	2	23,629,915	23,632,817		310	35324.21	5.79	69.27	Nuclear
*CtHDZIP10*	*Ct3T0173100.1*	1.80E-15	3	72,894,029	72,897,318		342	37449.09	8.49	59.09	Nuclear
*CtHDZIP11*	*Ct3T0197200.1*	4.00E-06	3	80,044,982	80,051,280		193	21194.45	4.36	61.74	Nuclear
*CtHDZIP12*	*Ct3T0265500.1*	1.70E-18	3	98,806,635	98,808,030		180	21281.53	5.29	73.58	Nuclear
*CtHDZIP13*	*Ct3T0289700.1*	3.50E-16	3	103,181,251	103,183,595		301	33727.88	8.75	63.33	Nuclear
*CtHDZIP14*	*Ct4T0121900.1*	3.20E-17	4	56,285,033	56,290,682		764	84554.60	5.63	49.16	Nuclear
*CtHDZIP15*	*Ct4T0143500.1*	4.00E-11	4	68,606,727	68,735,705		211	24382.64	9.30	55.18	Nuclear
*CtHDZIP16*	*Ct4T0176600.1*	5.50E-19	4	78,608,279	78,615,813		819	87798.40	5.53	49.62	Nuclear
*CtHDZIP17*	*Ct4T0230000.1*	2.60E-16	4	88,628,013	88,630,030		194	22177.87	7.61	47.13	Nuclear
*CtHDZIP18*	*Ct5T0120600.1*	7.20E-16	5	19,697,508	19,716,443		280	31858.07	9.05	56.18	Nuclear
*CtHDZIP19*	*Ct5T0133700.1*	1.10E-15	5	22,486,774	22,495,608		755	82805.40	6.31	48.52	Nuclear
*CtHDZIP20*	*Ct5T0237900.1*	2.60E-08	5	87,064,049	87,065,215		152	17494.54	5.97	44.73	Nuclear
*CtHDZIP21*	*Ct6T0010100.1*	5.10E-17	6	1,099,085	1,107,708		277	31369.31	4.99	39.54	Nuclear
*CtHDZIP22*	*Ct6T0122500.1*	2.90E-15	6	21,550,206	21,554,280		365	40277.93	5.83	58.36	Nuclear
*CtHDZIP23*	*Ct6T0219700.1*	3.20E-17	6	68,321,343	68,322,532		197	23327.99	5.62	66.25	Nuclear
*CtHDZIP24*	*Ct6T0259100.1*	1.30E-15	6	80,373,849	80,388,563		829	91010.73	6.55	43.98	Cytoplasmic
*CtHDZIP25*	*Ct6T0323300.1*	1.60E-17	6	92,031,967	92,034,458		310	34640.96	4.92	52.00	Nuclear
*CtHDZIP26*	*Ct7T0021700.1*	6.90E-16	7	2,685,224	2,686,766		294	33773.86	4.93	57.33	Nuclear
*CtHDZIP27*	*Ct7T0144000.1*	1.30E-18	7	29,702,043	29,711,968		843	90762.13	5.93	48.35	chloroplast
*CtHDZIP28*	*Ct7T0173500.1*	2.20E-18	7	44,350,603	44,355,482		737	81552.34	6.38	49.25	Nuclear
*CtHDZIP29*	*Ct8T0002600.3*	1.10E-17	8	331,627	336,748		208	24328.13	5.92	54.68	Nuclear
*CtHDZIP30*	*Ct8T0281500.1*	9.40E-19	8	68,963,845	68,966,140		236	26817.09	5.54	56.95	Nuclear
*CtHDZIP31*	*Ct8T0288300.1*	8.20E-18	8	70,750,084	70,755,618		707	77705.01	5.72	53.44	Nuclear
*CtHDZIP32*	*Ct9T0018300.1*	1.20E-15	9	2,049,916	2,058,693		832	91590.99	5.75	48.78	Cytoplasmic
*CtHDZIP33*	*Ct9T0020700.1*	4.10E-19	9	2,338,787	2,346,103		730	81449.15	6.07	46.34	Nuclear
*CtHDZIP34*	*Ct9T0062000.1*	1.30E-16	9	7,497,890	7,500,433		294	33704.01	4.82	66.84	Nuclear
*CtHDZIP35*	*Ct9T0291700.1*	1.60E-15	9	85,486,108	85,491,889		343	38451.00	6.18	62.73	Nuclear
*CtHDZIP36*	*Ct10T0040000.1*	5.10E-16	10	6,307,120	6,310,512		355	39483.26	9.00	74.22	Nuclear
*CtHDZIP37*	*Ct10T0172500.1*	1.90E-15	10	61,291,078	61,293,055		290	32092.98	8.42	63.96	Nuclear
*CtHDZIP38*	*Ct10T0183000.1*	5.60E-17	10	65,258,717	65,261,429		303	34582.14	5.00	45.97	Nuclear
*CtHDZIP39*	*Ct11T0012800.1*	1.90E-18	11	1,333,548	1,341,216		275	31142.01	4.66	60.58	Nuclear
*CtHDZIP40*	*Ct11T0022200.1*	5.70E-16	11	2,728,630	2,733,246		308	34065.84	8.61	51.6	Nuclear
*CtHDZIP41*	*Ct11T0063000.1*	5.90E-16	11	10,156,995	10,158,979		264	30357.97	5.88	51.97	Nuclear
*CtHDZIP42*	*Ct11T0164800.1*	2.10E-15	11	57,387,033	57,392,936		848	93289.39	5.96	45.89	chloroplast
*CtHDZIP43*	*Ct11T0198800.1*	9.10E-19	11	63,172,922	63,178,380		258	29213.77	6.23	53.74	Nuclear
*CtHDZIP44*	*Ct11T0211000.1*	1.30E-15	11	65,208,541	65,215,342		818	90097.42	6.04	51.28	chloroplast
*CtHDZIP45*	*Ct12T0151900.1*	3.90E-16	12	71,522,849	71,527,371		342	39858.93	5.36	65.52	Nuclear
*CtHDZIP46*	*Ct12T0152000.1*	2.80E-15	12	71,530,495	71,532,805		251	29202.57	6.64	60.93	Nuclear

### Phylogenetic analysis of HD-ZIP proteins in C. tinctorius

**Fig. 2:**
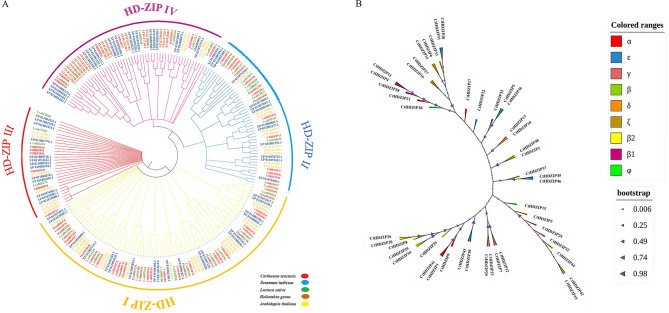
Classification of safflower HD-ZIP proteins based on sequence homology. **A** Circular phylogenetic tree of HD-ZIP genes in safflower, lettuce, sesame, sunflower, and Arabidopsis. **B** Phylogenetic tree of HD-ZIP genes of safflower in which subgroups in each HD-ZIP subfamily was determined with different colors

The homology search is a powerful tool for gene/protein classification. Comprehension of the similarity between the HD-ZIPs of safflower and other plant species is essential for investigating evolutionary processes and finding common ancestors. The HD-ZIP genes were classified into four subfamilies, HD-ZIP I-IV, based on their Arabidopsis counterparts (Supplementary Table S3), that is fairly reflected in the circular phylogenetic tree constructed between *C. tinctorius* and the other studied plant species (Fig. 2A). These valuable findings can be utilized for predicting function and relationship of safflower HD-ZIPs genes. HD-ZIP proteins in our study showed classification patterns similar to *A. thaliana* and *Sesamum indicum* that were identified in previous research [32, 62].

Next, to determine subgroups within each of the main HD-ZIP groups, a phylogenetic analysis was utilized using *A. thaliana* subgroups as template **(**Fig. 2B**)**. The HD-ZIP I group had seven clades consisting of α, β1, β2, ε, γ, δ and φ. The HD-ZIP II is divided into α, β, γ, δ and ε subgroups. The HD-ZIP III consists of β and γ clades and the fourth HD-ZIP group includes ε, ζ, and δ. As seen, the ζ subgroup existed only in the HD-ZIP IV. In the HD-ZIP I subfamily the highest homology was recorded between safflower genes *CtHDZIP20* and *CtHDZIP26* with Arabidopsis gene *AT3G01470* (HB-1, β2 subgroup). The *CtHDZIP10* gene in HD-ZIP II group had the highest homology with *AT3G60390* (HAT3, δ subgroup), and in the HD-ZIP III subfamily the highest homology was between the *CtHDZIP32* and *AT1G52150* (HB-15,γ subgroup). In HD-ZIP IV the highest homology was between *CtHDZIP14* and *AT4G21750* (ATML1, δ subgroup). Genes *CtHDZIP1*, *CtHDZIP9*, *CtHDZIP21*, and *CtHDZIP41* belonged to α clade in HD-ZIP I group. The largest numbers of safflower HD-ZIP genes belonged to subgroups of ε (21%) and γ (17%), while the lowest numbers belonged to φ and ζ subgroups (4.2%). Subgroups of α and β2 allocated 10.6% and 12.7% of safflower HD-ZIP genes, respectively.

### Conserved motifs structure of HD-ZIP proteins in C. tinctorius

**Fig. 3:**
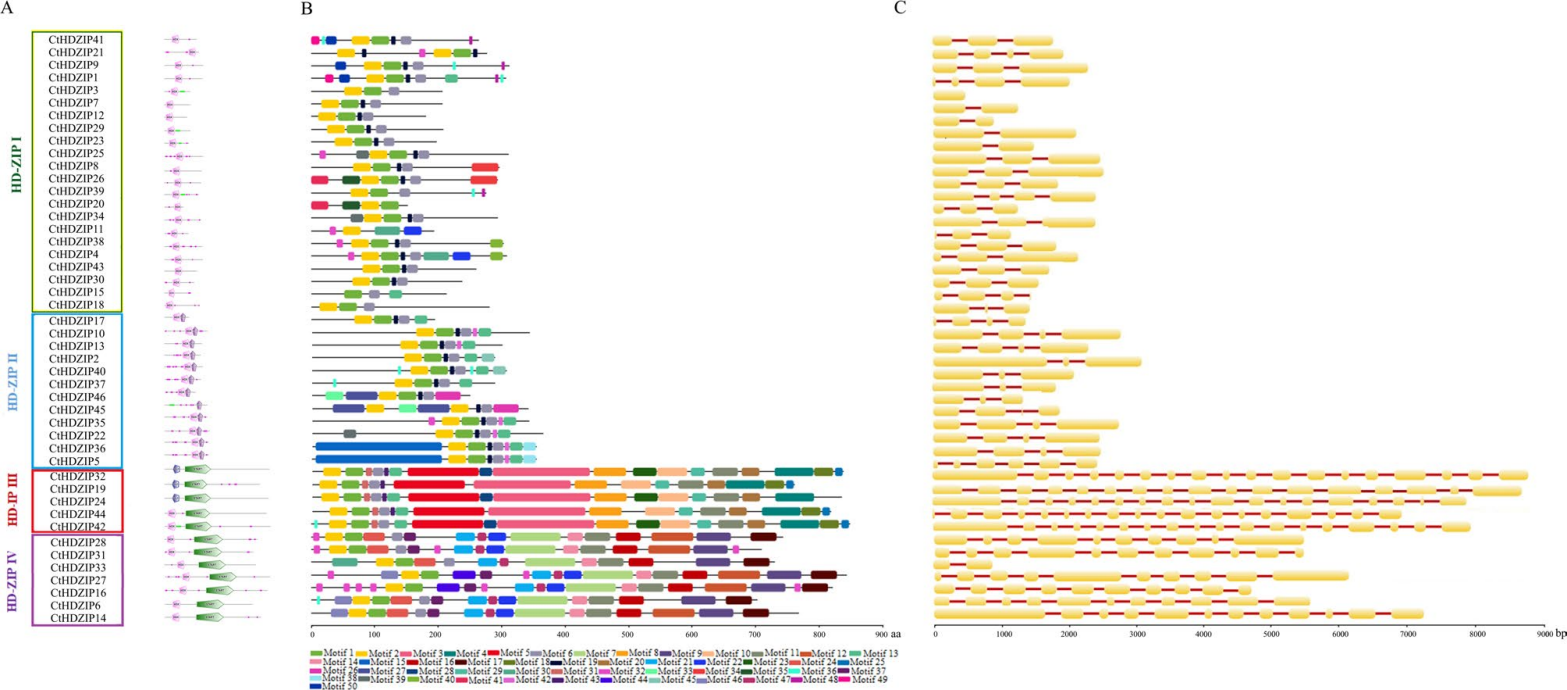
Structure of HD-ZIP gene family in safflower. **A** The occurrence and position of HD-ZIP specific domains in four safflower HD-ZIP groups. **B** The predicted motifs by MEME in safflower HD-ZIP members. **C** The exon-intron structure of 46 safflower HD-ZIP genes. Yellow color displays the exons and red color shows the introns

Analysis of occurrence of known domains in 47 safflower HD-ZIP protein sequences showed that all groups of safflower HD-ZIPs had conserved motifs that can encode HD (HOX) and LZ domains (Fig. 3A). The domain structure of HD-ZIP I sequences is consisting homeodomain (HD/HOX, PF00046) and HALZ (PF02183). However, in *CtHDZIP21* gene, HD and LZ domains are located in C-terminal, unlike other members of the HD-ZIP I subfamily (Fig. 3A). This suggested segmental inversion has occurred in this gene.

The HD-ZIP II in addition to conserved homeodomain (HD/HOX) and HALZ sequences has CPSCE region in the carboxy terminal. The HD-ZIP III contains the HD, LZ, START, and MEKHLA (PF08670). The HD-ZIP IV members have HD and START domains. The achieved results might indicate the specific and similar function of members of each group. In the basis of the results of SMART and Pfam analyses, domain pattern in each of the HD-ZIP groups (I-IV) of safflower is completely distinct. The HD and LZ domains present in all of the HD-ZIP groups of safflower. It is worthy to point out that the MEKHLA domain is highly conserved among plant species and is also found in some bacterial proteins, suggesting an ancient evolutionary origin [63]. This domain is an exclusive feature of HD-ZIP III subfamily, and is not present in other HD-ZIP subfamilies [64, 65]. The domain shares significant sequence and structural similarity with the PAS (Per-ARNT-Sim) domain, a well-known sensory module found across all kingdoms of life. The domain may allow HD-ZIP III transcription factors to integrate environmental cues into developmental programs, adjusting gene expression in response to changing conditions [63, 66]. Indeed, the MEKHLA domain functions as a negative regulator, modulating the protein’s transcriptional activity and its ability to form dimers with other proteins [66].

Conserved motif structures and their diversification using MEME motif discovery tool (https://meme-suite.org/meme/) were predicted which was resulted to the recognition of 50 distinct motifs **(**Fig. 3B; Supplementary Fig. S1). The longest motifs (200 aa) are motifs 3 and 15. The most prevalent motifs are motifs 1 and 2 which exist in all members of four HD-ZIP groups. These two motifs almost span the homeodomain and LZ domain. Most members closely related in the phylogenetic tree of each subfamily have prevalent motif patterns, demonstrating similarity of function in the same subfamily. The motif 19 is found in majority of all members of HD-ZIP I and II groups, and motif 11 is found in both HD-ZIP III and IV members. Most putative motifs in subfamilies III and IV are distributed throughout the protein sequence. Some motifs are preferentially distributed across specifc HD-ZIP groups. For instance, the conserved motifs 3 and 4 are only identified in HD-ZIP III genes. It seems the motif 4 spans the MEKHLA domain. The HD-ZIP III group`s specificity of MEKHLA domain is consistent with previous studies [30, 32–34]. Another prominently distributed motifs in HD-ZIP III group are motifs 8, 10 and 13. The motifs 7, 9, 14 and 16 are specific to HD-ZIP IV group.

To comprehend gene structure and diversity of exon-intron counts, the Gene Structure Display Server (GSDS) was utilized (Fig. 3C). Number of exons (CDS) was variable between 2 and 18. The largest number of exons was in the HD-ZIP III group (*n* = 18) and the lowest was in HD-ZIP I group (*n* = 2). Furthermore, the lowest and the largest number of introns, respectively, were observed in HD-ZIP I (*n* = 1) and HD-ZIP III (*n* = 17). The HD-ZIP II genes have 3, 4 or 6 exons. There are 9–11 exons in the genes of HD-ZIP IV group.

### Chromosomal location and gene duplication analysis

**Fig. 4:**
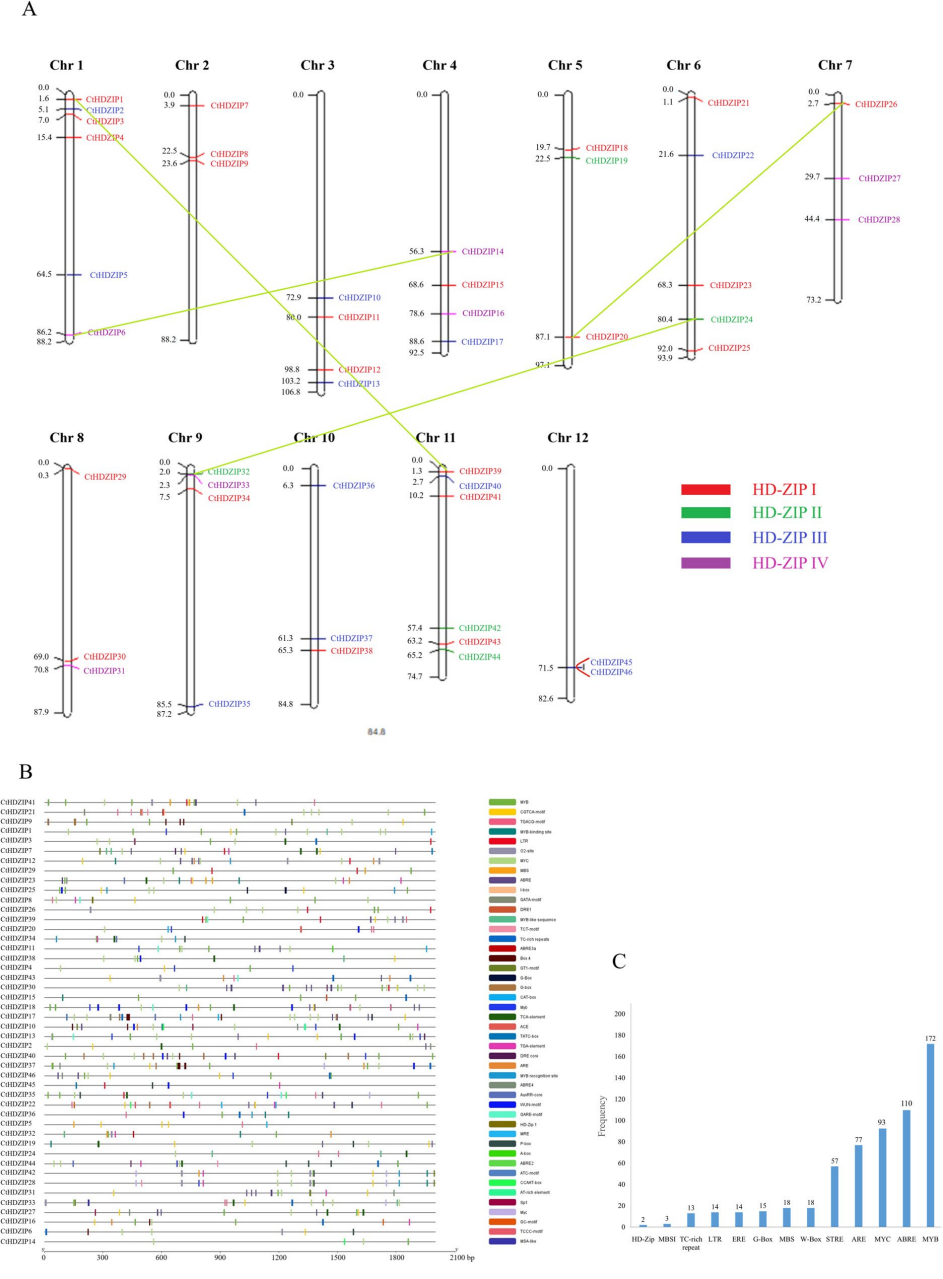
Chromosomal locations and structural features of safflower HD-ZIP promoters. **A** Map locations of HD-ZIP genes across 12 safflower chromosomes. The gene’s name is on the right side and the physical position (Mbp) is indicated on the left side. Segmental duplications are connected with light-green straight lines and tandem duplications are connected with orange curved lines. **B** Ocurrence of different *cis*-elements in promoter sequences of safflower HD-ZIP genes. **C** Frequency of drought- and hormone-responsive *cis*-elements in promoter sequences of safflower HD-ZIP genes

The chromosomal location of 47 HD-ZIP genes of safflower was investigated to predict gene differentiation and duplication (Fig. 4A). The chromosomal position was achieved based on the safflower genome annotation available at https://safflower.scuec.edu.cn/. In the Fig. 4A, HD-ZIP I, II, III, and IV genes are represented in red, blue, green and purple colors, respectively. As seen, the HD-ZIP genes are distributed on all 12 chromosomes of safflower. The highest number of HD-ZIP genes are located on chromosomes 1 and 11 (*n* = 6 on each) and the lowest number of HD-ZIP genes are positioned on chromosome 12 (*n* = 2). The genes of the HD-ZIP III group are only located on chromosomes 2, 5, 6, 9 and 11. Chromosome 12 harbors only HD-ZIP II genes.

In this research we identified 47 HD-ZIP genes distributed across all 12 chromosomes of safflower. Although this number is fewer than their number in some species [e.g., *Actinidia chinensis* [67], *Pyrus bretschneideri* [68] and *Brassica napus* [69], it is greater than their number in other species [e.g., *Dendrobium officinale* [22], *Prunus persica* [30], and *Hippophae rhamnoides* [70]. This shows that gene duplication events may have occurred during the evolution of safflower genome. Gene duplication is known to contribute to gene family expansion and the emergence of novel functions [71]. We identified 18 gene pairs in the *C. tinctorius* genome among *CtHD-ZIP* genes (Supplementary Table S4). One pair (*CtHDZIP45/HDZIP46*) resulted from tandem duplication, and four pairs (*CtHDZIP24/CtHDZIP32*,* CtHDZIP6/CtHDZIP14*,* CtHDZIP1/CtHDZIP41*,* and CtHDZIP20/CtHDZIP26*) resulted from segmental (genome-wide) duplications. Although tandem duplication and large fragment replication are accounted to primarily contribute to gene family expansion [72], our results indicates the HD-ZIP family of safflower has mainly extended by segmental (genome-wide) duplication events, similar to what observed for soybean [73], kiwifruit [67], peach [30] and sea buckthorn [70]. Segmental duplication is believed to be associated to the slow evolutionary rates, thus reinforcing the high conservation of the HD-ZIP gene family over time [74].

### Identification of cis-acting elements in HD-ZIP gene family promoters

The promoters are important elements in gene expression patterns in different abiotic stresses, therefore their identification and discovery of *cis*-elements to find the mechanisms of response to abiotic stresses is necessary [75, 76]. Advances in transcriptional expression led to the identification of trans-acting proteins and various compensatory factors in the promoter regions of stress-inducible genes [77]. Analysis of HD-ZIP promoter *cis*-elements revealed that eukaryot-specific elements TATA-box and CAAT-box have the highest numbers in these promoter sequences (Fig. 4B). These cis elements are key regulatory elements of gene expression in plants. TATA-box serves as the binding site for the TATA-binding protein (TBP), a subunit of the transcription factor TFIID [78]. It is found in many highly expressed genes and is a key determinant of transcription efficiency and specificity in plants. It especially contributes to light-dependent gene expression, modulating transcription in response to environmental cues [78, 79]. Intrestingly, it was reported that some members of HD-ZIP I group selectively interact with TBP [80]. CAAT-box acts as a binding site for CAAT-binding transcription factors (e.g., NF-Y) and often is involved in regulating basal transcription levels and can influence promoter strength [81]. It helps stabilize the pre-initiation complex and facilitates RNA polymerase binding, thus enhancing transcription initiation. CAAT-boxes are less common in genes expressed ubiquitously and tend to be associated with regulated or tissue-specific expression [82, 83].

The putative regulatory elements related to phytohormones (Table 2) and abiotic stresses were also discovered in the promoters of HD-ZIP genes (Fig. 4C; Table 3). The ABRE (*n* = 110) sequence is involved in the promoter of genes regulating drought tolerance that are important for inducing tolerance to the stresses faced by polygenic families. ABRE and TCA cis-elements are regulatory and play an important role in responding to biotic and abiotic stresses in plants [20]. The MYB (*n* = 172) and MYC (*n* = 93) transcription factor cis-elements are frequent in promoters of HD-ZIP genes which bind to the promoter of the target genes under abiotic stresses. Members of MYB and MYC families are involved in the activation ABA pathway in response to drought stress and are regulated by inducing the genes of tolerance to drought stress [84]. The number of MBS (MYB binding sites involved in drought-inducibility) with CAACTG consensus sequence was 14, most of them are found in promoters of the HD-ZIP I and II group. Members of HD-ZIP I and II are involved in response to drought stress [68, 85, 86]. We found CAATNATTG *cis*-element (BS1/BS2) in promoter of *CtHDZIP8*, *CtHDZIP40* and *CtHDZIP5* genes belonging to HD-ZIPs I & II subfamilies. These elements were firstly reported by Tron et al. [87] in the promoter of HD-ZIPs I & II genes and discussed by Harris et al. [85].

**Table 2:** Identified *cis*-elements related to hormone response in promoter sequences of safflower HD-ZIPs

Cis-element	Sequence	Function
ABRE	GACACGTGGC ACGTG TACGTGTC CACGTG GCAACGTGTC AACCCGG	Abscisic acid responsiveness
ABRE3a	TACGTG	Abscisic acid responsiveness
ABRE4	CACGTA	Abscisic acid responsiveness
CGTCA-motif	CGTCA	MeJA-responsiveness
TGACG-motif	TGACG	
TATC-box	TATCCCA	Gibberellin-responsiveness
GARE-motif	TCTGTTG	
P-box	CCTTTTG	
TGA-element	AACGAC	Auxin responsiveness
AuxRR-core	GGTCCAT	Auxin responsiveness
TCA-element	CCATCTTTTT TCAGAAGAGG	Salicylic acid responsiveness

**Table 3:** Identified *cis*-elements related to abiotic stresses in promoter sequences of safflower HD-ZIPs

*Cis*-element	Sequence	Function
MYB	TAACCA	Response to stress
	CAACAG	
	TAACTG	
	CAACTG	
	CCGTTG	
	CAACCA	
MBS	CAACTG	MYB binding site involved in drought-inducibility
MRE	AACCTAA	MYB binding site involved in light responsiveness
MBSI	aaaAaaC(G/C)GTTA	MYB binding site involved in flavonoid biosynthetic gene regulation
MYC	CATTTG CATGTG TCTCTTA	Response to stress
DRE core	GCCGAC	dehydration responsive
LTR	CCGAAA	low-temperature responsiveness
TC-rich repeats	ATTCTCTAAC GTTTTCTTAC	defense and stress responsiveness
G-Box	CACGTG TACGTG tgACACGTGGCA ACACGTGGC CACGAC GCCACGTGGA	light responsiveness
ACE	CTAACGTATT GACACGTATG	light responsiveness
GT1-motif	GGTTAAT	light responsive element
O2-site	GATGA(C/T)(A/G)TG(A/G) GATGATGTGG GTTGACGTGA GATGACATGG	zein metabolism regulation
CCAAT-box	CAACGG	MYBHv1 binding site
GT1-motif	GTGTGTGAA	light responsive element
Sp1	GGGCGG	light responsive element
WUN-motif	AAATTTCCT AAATTACTA AAATTTCTT TTATTACAT	wound-responsive element
BS1/BS2	CAATNATTG	differentiation of the palisade mesophyll cells

### Synteny analysis in HD-ZIPs family genes in Carthamus tinctorius

**Fig. 5:**
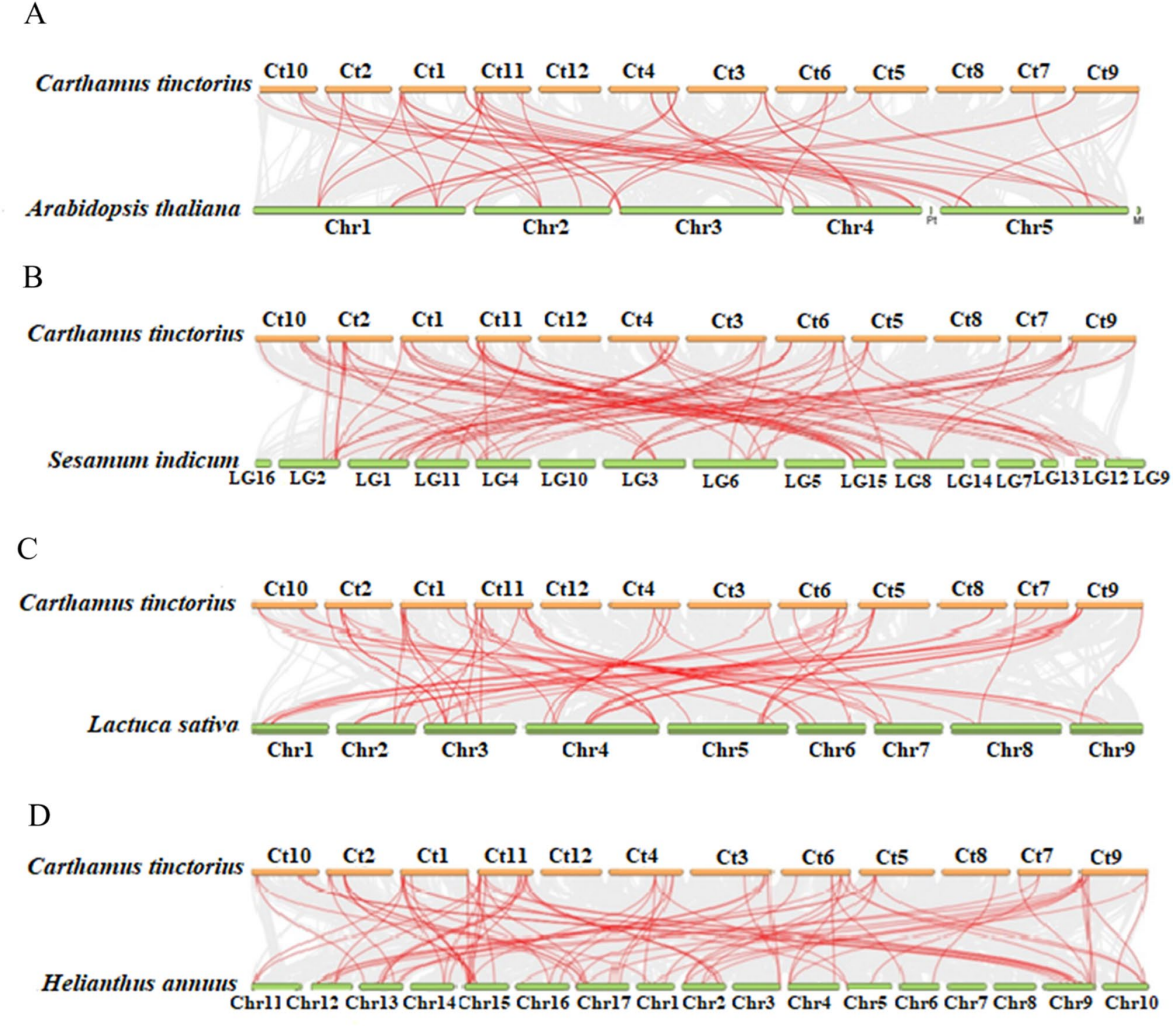
Collinearity relationship of the HD-ZIP genes among safflower and other plants. **A **Gene collinearity between safflower and Arabidopsis. **B** Gene collinearity between safflower and sesame. **C** Gene collinearity between safflower and letuce. **D** Gene collinearity between safflower and sunflower

The collinearity relationship of safflower HD-ZIP genes and four other plant species was investigated to obtain the evolutionary forces that drove the expansion or contraction of *C. tinctorius* HD-ZIP gene family (Fig. 5). The analysis showed that there were 44 collinearity pairs of HD-ZIP genes between safflower and Arabidopsis. However, the collinearity of HD-ZIP gene pairs was higher with other plant species. Collinearity pairs between safflower and lettuce enumerated 57, and between safflower and sesamum enumerated 66. The results of collinearity analysis between safflower and sunflower revealed 88 collinearity pairs of HD-ZIP genes. According to our synteny analysis, safflower showed a closer genetic relationship with members of Astraceae family (sunflower, sesame and lettuce), and since the lowest rate of collinearity was observed between safflower and Arabidopsis, Arabidopsis could be considered as an outgroup.

### Divergence analysis of duplicated genes

The thirty four HD-ZIP genes were classified in the closest branches in the phylogenetic tree (Fig. 2A), indicating they probably were under similar evolution drivers. The DNA sequence intra-group alignment of the HD-ZIP genes revealed five gene pairs with sequence identities higher than 60%, and these pairs showed a high covariance relationship following analysis using MCScanX (Table 4). We calculated the Ka (nonsynonymous substitutions)/Ks (synonymous substitutions) ratio for defining the type of selection pressure on duplicated genes. Ka/Ks ratio expresses the degree of evolutionary change [88]. The Ka/Ks ratio more than 1 suggests that positive selection affected duplicated genes, and the Ka/Ks ratio less than 1 suggests that purifying selection affected duplicated genes. The Ka/Ks ratios are shown in Table 4. The Ka/Ks for two pairs of HD-ZIP genes (*CtHDZIP24/CtHDZIP32* and *CtHDZIP1/CtHDZIP41*) was higher than 1, and for three pairs (*CtHDZIP6/CtHDZIP14*,* CtHDZIP45/CtHDZIP46* and *CtHDZIP20/CtHDZIP26*) was lower than 1. This analysis indicates that the former pairs have been under the positive selection, and the later ones have been under the purifying selection. Our results indicate the duplicated gene pairs were under both positive and purifying selection in safflower. Meanwhile, it was reported that the HD-ZIP genes in soybean [73] and kiwifruit [67] have been exclusively under the purifying selection. Positive selection (also called directional or diversifying selection) favors mutations or alleles that increase an organism’s fitness. These beneficial mutations tend to increase in frequency over generations because they confer an advantage, such as better adaptation to the environment. For example, mutations that improve disease resistance or reproductive success are positively selected [89]. In contrast, purifying selection (also known as negative selection) acts to remove deleterious or harmful mutations from the population. It reduces the frequency of alleles that decrease fitness, thereby preserving the integrity and function of essential genes. This process leads to the conservation of gene sequences over time and stabilizes populations by purging detrimental genetic variants [90].

**Table 4:** Ka/Ks ratios for five pairs of safflower duplicated HD-ZIP genes. The group of HD-ZIP is presented in parentheses

Duplicated gene 1	Duplicated gene 2	Ka	Ks	vKa	vKs	Ka: Ks	Duplication type
*CtHDZIP1 (I)*	*CtHDZIP41 (I)*	0.4465	0.273	0.0019	0.0029	1.6357	Segmental
*CtHDZIP20 (I)*	*CtHDZIP26 (I)*	0.1482	0.2189	0.00073	0.0032	0.677	Segmental
*CtHDZIP45 (II)*	*CtHDZIP46 (II)*	0.0046	0.0439	1.06E-05	0.00039	0.1047	Tandem
*CtHDZIP24 (III)*	*CtHDZIP32(III)*	0.3656	0.2304	0.00042	0.0007	1.5865	Segmental
*CtHDZIP6 (IV)*	*CtHDZIP14 (IV)*	0.1614	0.6345	0.00018	0.0028	0.2544	Segmental

*vKa* variance for Ka, *vKs* variance for Ks

Duplication is a major source of genetic diversity in different species and causes new functions for genes [91]. One of the consequences of gene duplication is that functional redundancy can lead to the amplification of gene families [92]. During evolution, duplicated genes can be maintained by selection in gene sequences. The maintenance of duplicated genes in genomes is due to crucial functions of genes in response to environmental stresses [93].

### Three-dimensional structures of HD-ZIP proteins in C. tinctorius

To investigate the structural characteristics and undrestand the function of HD-ZIP proteins in safflower, three-dimensional models of HD-ZIP protein families were constructed (Fig. S2). The results indicate that the three-dimensional structures of HD-ZIP proteins of members of each subfamily are similar. The 3-D structures of HD-ZIP I & II groups are more similar with eachother and it is meaningfully different than that of HD-ZIP III & IV groups. It sounds like HD-ZIP III & IV structures are more similar and their members are distinct in protein structure from members of HD-ZIP I & II groups.

### Subcellular localization of HD-ZIP proteins in Carthamus tinctorius

The subcellular position of proteins helped predict their function. The WoLF PSORT results demonstrated the majority of the HD-ZIP proteins of safflower are localized to the cell nucleus and some of them are localized to the cytoplasm and chloroplast. Given that HD-ZIP proteins are transcriptional factors and their original location is the nucleus, our results are consistent with this fact. The previous study confirmed that the localization of HD-ZIPs in nucleus is directly relative to their function and regulation of downstream gene expression by binding to gene promoters [86, 94]. For example, the gene *CtHDZIP37* is safflower homolog of *HAT22* in Arabidopsis, and similar to members of the HD-ZIP II group is localized to the nucleus [95].

### Gene ontology enrichment and protein-protein interaction network of HD-ZIP genes

The transcription factors can act as a tool for plant tolerance/resistance and persistence of yield in drought stress [95]. The HD-ZIP proteins have been evidenced to be very curtailed and unique in many biological and physiological processes such as growth and developmental processes, and response to drought stress in plants [68, 86]. However, no report on the function of HD-ZIP proteins has been reported in safflower.

Over representation analyses (ORA) including gene ontology (GO) and KEGG analysis were performed using a cut-off value of *P* < 0.05. No KEGG pathway was enriched for HD-ZIPs based on g: Profiler tool. However, GO analysis revealed 194 biological processes (BP), 17 molecular functions (MF), and 8 cellular components (CC) for HD-ZIP genes (Fig. S3 A). The analysis confirmed DNA-binding transcription factor activity (GO:0003700) of all HD-ZIPs with high confidence (adjusted p value of 4.81E-25) and nucleus transportation (GO:0005634, adjusted p value of 2.74E-14). The majority of HD-ZIPs were enriched for involvement in biological processes (Fig. S3 B). According to the GO analysis, the gene *CtHDZIP3* (*HB-40*, HD-ZIP I subfamily) involves in stress response (GO:0006950), and genes *CtHDZIP7*, *CtHDZIP12*, *CtHDZIP29* and *CtHDZIP23* (all are safflower homologs of *HB-7*) are involved in response to ABA (GO:0009737), response to stimulus, response to chemical, response to water (GO:0009415) and response to water deprivation (GO:0009414). Studies showed that HD-ZIP proteins regulate the expression of responsive genes under drought stress through ABA and MAPK signaling [86, 96].

**Fig. 6:**
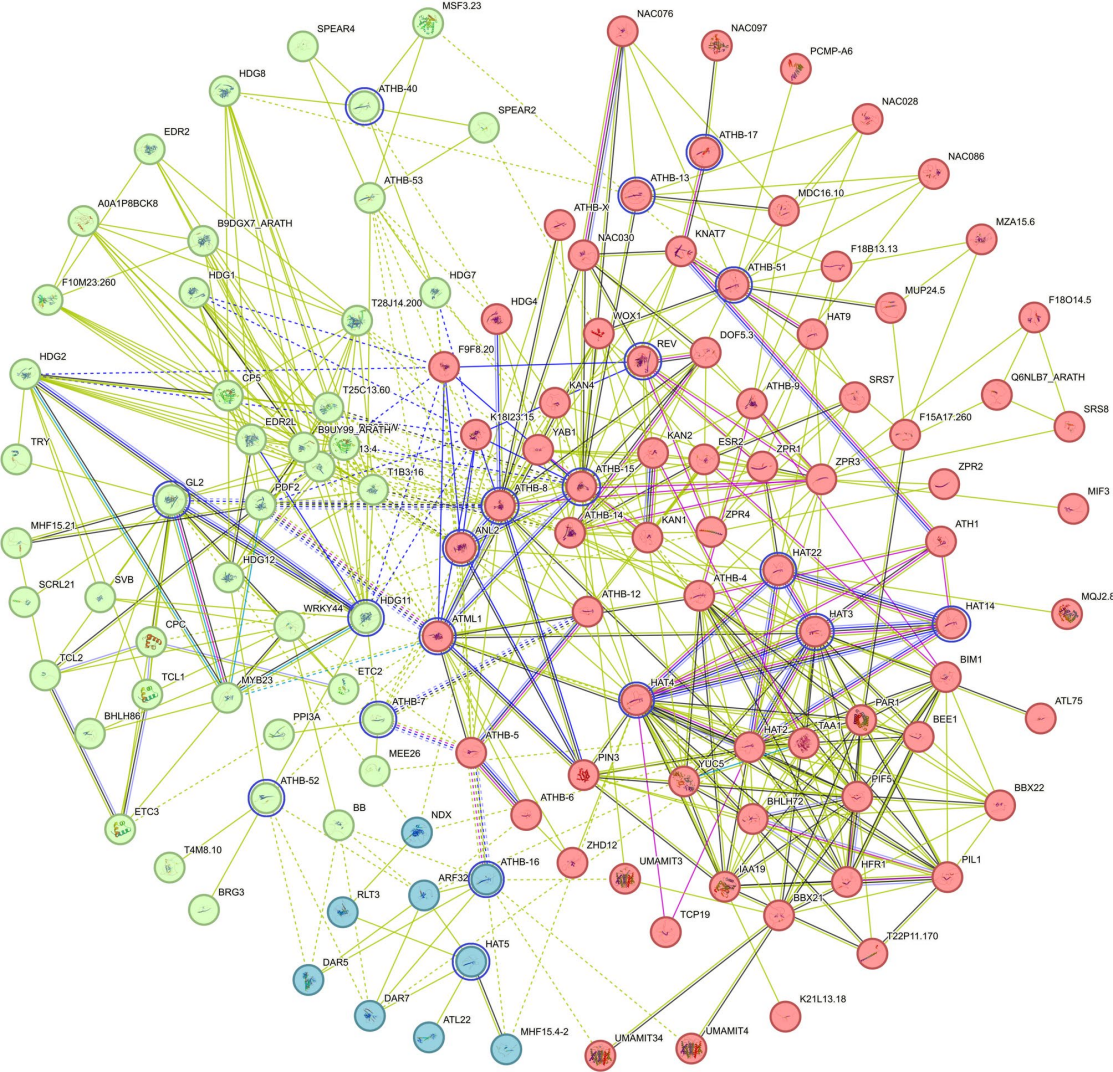
Protein-protein interaction network of safflower HD-ZIP genes from the perspective of *A. thaliana* homologous unigenes. Three gene modules are recogonized, marked with different colorings (HZ-blue, HZ-green and HZ-red). Proteins in each module are connected with solid lines, but proteins in different modules are connected with dotted lines. Blue circles show the safflower homologs of Arabidopsis HD-ZIPs. Less valid connections are shown with yellow lines. Annotations and descriptions of the network members are represented in Supplementary Table S4

PPI network of HD-ZIP gene family was constructed using STRING for all safflower homologs of 19 Arabidopsis unigenes (Fig. 6). This analysis reveals three gene modules (viz. HZ-blue, HZ-green and HZ-red modules), each of wich contains at least two safflower homologs of Arabidopsis HD-ZIP unigenes. As seen in the figure, HD-ZIP subfamily members are randomly distributed in these 3 modules. 2, 5 and 12 safflower homologs of HD-ZIP unigenes were clustered in the three modules, respectively. In HZ-red module, the interaction among HAT3, HAT4, HAT14 and HAT22, all belonging to HD-ZIP II subfamily, is of high degree of cross-validation supports. GL2 (GLABRA 2) in HZ-green module interacts with MHF15.21 (putative B-type cyclin) and MYB23 transcription factor that probably are required for trichome initiation and formation (see Supplementary Table S3 for annotation and description of the network members). The GL2 also interacts with HDG11 and two TFs (MYB23, and WRKY44) and with a member of HZ-red module, ATHB-8. ATHB-15 is a hub gene in HZ-red module, interacting with ATHB-13, Dl3290W (polyketide cyclase/dehydrase and lipid transport superfamily protein), ZPR3 (protein LITTLE ZIPPER 3, involved in SAM development and lateral organ patterning), ANL2, ATML1, two PLAC8 family proteins (F9F8.20, K18I23.15) and three TFs (DOF5.3, NAC030 and NAC076). AtHB-7, although placed in HZ-green module, shows valid interactions with members of HZ-red module including ATHB-5 and ATHB-12. ATHB-8 in HZ-red module, in addition to the interaction with two members of HZ-green module (GL2 and PDF2), interacts with other members of HZ-red module (PIN3, ZPR3, HAT2, K1812315, HDG4, ATHB-X, DOF5.3 and NAC030). As can be seen from these interactions, some transcription factors from other gene families play a role in regulating biological processes in harmoy with members of HD-ZIP gene family.

Although most important interactions of safflower homologs of HD-ZIP unigenes were determined via constructing PPI network in this study, thers is a clear gap yet in interactions of three of important HD-ZIP unigenes including HAT5, HB-16 and HB-40, all belonging to HD-ZIP I subfamily. Therefore, the future research should focus on determining their interactions.

### Expression profile of HD-ZIP genes of C. tinctorius under drought stress

**Fig. 7:**
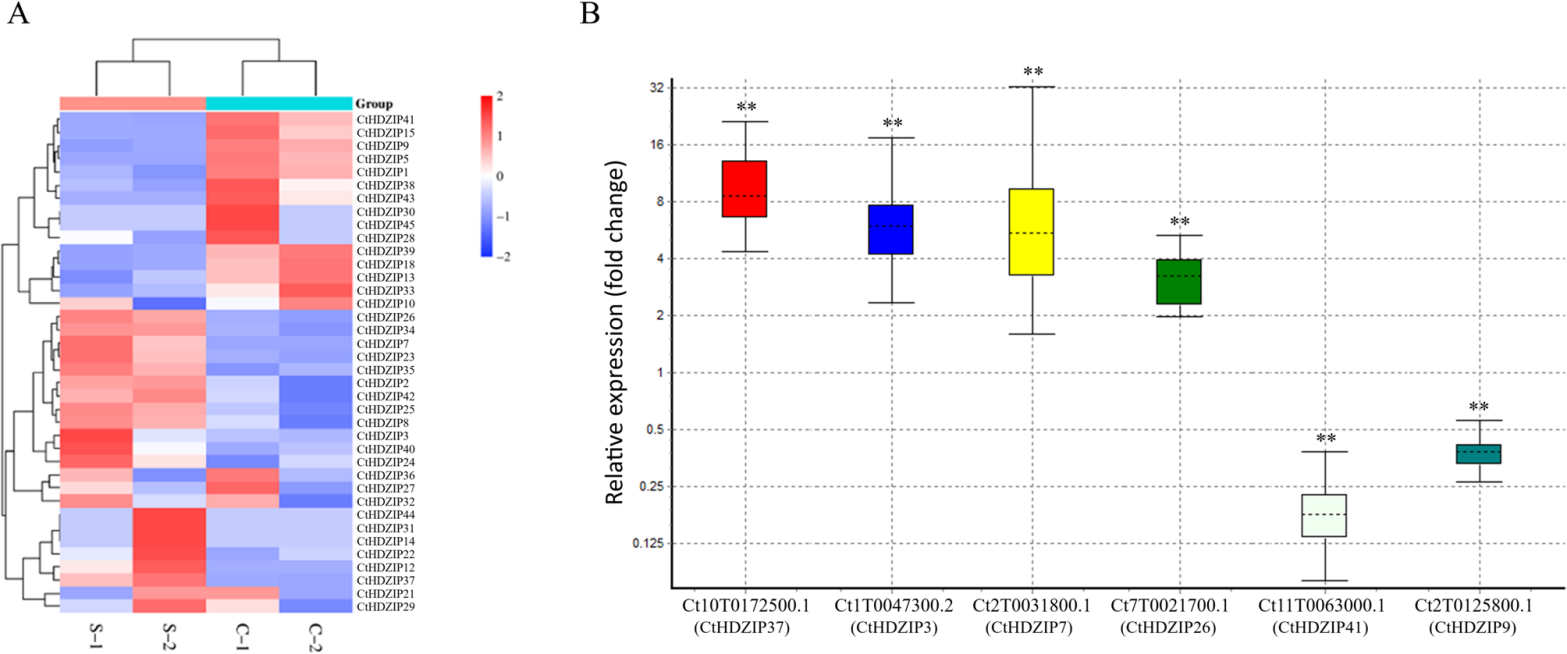
Expression pattern of safflower HD-ZIP genes under drought stress at vegetative stage. **A** A heatmap representation of the expression pattern of HD-ZIP genes in safflower under drought stress in RNA-seq assay. C-1 and C-2 are control non-stressed samples, and S-1 and S2 are drought-stressed samples. The different colors show different expression levels. Red boxes indicate upregulated and blue boxes indicate downregulated genes. **B** The relative changes in the gene expression of HD-ZIPs in safflower seedlings in response to drought stress revealed by real-time qRT-PCR. ** indicates significance at *P* < 0.01

To perceive the effect of drought stress on the expression pattern of HD-ZIP genes in young safflower seedlings, transcriptional levels of 47 HD-ZIP genes were investigated using the RNA-seq assay (Fig. 7A). Our results indicate that the highest number of drought-responsive genes belong to HD-ZIP I group. The genes *CtHDZIP7*, *CtHDZIP12*, *CtHDZIP29* and *CtHDZIP23* were upregulated under drought stress; these gene are orthologs of *AtHB7* (*AT2G46680*) belonging to the γ subgroup of HD-ZIP I group. The *CtHDZIP3*, ortholog of *AtHB40* (*AT4G36740.1*), belonging to δ clade of HD-ZIP I group, was upregulated in drought stress. *CtHDZIP38* is ortholog of *AtHB16* (*AT4G40060*, HD-ZIP I group) which was downregulated under drought stress. The previous studies showed that the *AtHB16* gene was downregulated in response to drought stress in roots and leaves of Arabidopsis [85, 97]. It was reported that *AtHB16* acted as a negative regulator of cell expansion [98]. Intrestingly, *LlHOX6* (a member of HD-ZIP I group) antagonizes homeobox protein *LlHB16* to attenuate basal thermotolerance in lily [99]. Expression of *CtHDZIP9*, orthologous to *HB13* (*AT1G69780*), belonging to α subgroup of HD-ZIP I was downregulated under drought stress. The members of the HD-ZIP I are key players in response to environmental stresses [80, 86]. Furthermore, the *CtHDZIP15*, ortholog of *HB52* (*AT5G53980*), belonging to Φ subgroup of HD-ZIP I group, was downregulated under drought stress. *CtHDZIP26*, ortholog of *HAT5*/*HB-1* (*AT3G01470*) from β2 subgroup of HD-ZIP I was upregulated under drought stress (Fig. 7A).

From the above-mentioned genes, seven genes in HD-ZIP I subfamily showed significant differential expression under drought stress. The genes *CtHDZIP3* (*HB40*) (LFC = 3.39, FDR = 0.001374), *CtHDZIP7* (*HB7*) (LFC = 6.51, FDR = 4.29E-26), *CtHDZIP23* (*HB7*) (LFC = 2.44, FDR = 0.0396), and *CtHDZIP26* (*HAT5/HB-1*) (LFC = 3.85, FDR = 3.19E-09) were significantly upregulated under drought stress, and the genes *CtHDZIP41* (*HB13*) (LFC=−5.44, FDR = 0.001124), *CtHDZIP9* (*HB13*) (LFC=−2.23, FDR = 1.46E-07) and *CtHDZIP15* (*HB52*) (LFC=−1.52, FDR = 0.03136) were significantly downregulated under drought stress.

In HD-ZIP II subfamily two genes showed differential expression: *CtHDZIP37*, homolog of *HAT22* (*AT4G37790*), was significantly upregulated in drought stress (LFC = 4.48, FDR = 0.000902), and *CtHDZIP5*, ortholog of *HAT14* (*AT5G06710*), was downregulated but wasn’t significant (LFC=−2.71, FDR = 0.7494). The genes in two other subfamilies (III and IV) weren’t sigficantly expressed under drought stress (Fig. 7A). Expression of the *HAT22* gene was increased in Arabidopsis when exposed to drought stress [100]. Previous studies demonstrated that *AtHB7*, *AtHB12*, *HaHB4*, *OsHOX6*, *OsHOX22*, and *OsHOX24* [40, 85], were upregulated under water deficiency conditions in young tissues of Arabidopsis [101] and regulated by drought stress and ABA [97]. Based on RNA-seq results, the HD-ZIP genes involved in response to drought stress in safflower belong to HD-ZIP I and II subfamilies.

Water deficit damages plant growth and development in safflower [102], and members of the HD-ZIP I group, particularly safflower homologs of *AtHB7*, respond to it via altered expression. *AtHB7* is upregulated in drought and salt stresses in meristem, root tips and flowers [103]. RNA-seq assy showed that the *AtHB7* in *A. thaliana* and its sunflower orthologs, *HaHB4* and *HaHB11*, were upregulated in water deficit stress [16, 103, 104]. *AtHB7* along with *AtHB12* regulate root growth in response to Aluminum stress [105]. The gene improves drought tolerance in ice plant through ABA signaling pathway [106], and indirectly involves in positive regulation of drought and salt stress tolerance in cotton [107]. *AtHB13* functions as a positive regulator of the drought-responsive transcription factor *JUB1*. It enhances drought tolerance when overexpressed, acting at later developmental stages and under drought stress conditions [108]. This involves direct binding to the *JUB1* promoter and nuclear localization consistent with its role as a transcriptional regulator.

It was reported that *AtHB40* is upregulated in response to ABA and drought stress [109] as well as in response to salt and drought stress [98]. Recent studies unravel the function of *AtHB40* in different processes including primary root length and gravitropism [110], high temperature, osmotic and salinity stresses [111], and water deficit in sunflower [112]. Although direct functional studies on *AtHB40*’s role in drought tolerance are less detailed than for *AtHB13* [108], its induction by ABA and stress conditions suggests it participates in the regulatory network that enhances drought tolerance. This likely involves modulation of gene expression downstream of ABA signaling, contributing to improved plant adaptation to water deficit.

The *CtHDZIP26* (safflower homolog of *AtHB1/HAT5*) is upregulated in safflower under drought and involves in response to stress (GO:0006950), response to stimulus (GO:0050896), response to abiotic stimulus (GO:0009628), stress response (GO:0006950) and response to salt stress (GO:0009651). The expression of *AtHB1* was upregulated in light stress and downregulated in salt and temperature stresses [113]. In our research, according to GO analysis, *CtHDZIP26* involves in response to light stimulus (GO:0009416) and developmental process (GO:0032502). Similar results were reported by Li et al. [29] for *AtHB1*. Reportedly, the members of the HD-ZIP I group are important for regulating the response to drought and salt stresses in rice and maize [45, 74, 114]. The *HaHB1* gene in sunflower (*H. annuus*) and its *Arabidopsis* homolog *AtHB13*, are induced in response to drought and cold stresses [114]. Two safflower genes (*CtHDZIP41* and *CtHDZIP9*) which are homologs of *HB13* (HD-ZIP I group), were downregulated under drought stress. *AtHB13* is implicated in sucrose signaling pathways and responses to abiotic stresses such as freezing, drought, and salinity. It influences gene expression related to these stress responses, possibly helping plants adapt to adverse environmental conditions [115]. The overexpression of *HaHB1* in sunflower and *AtHB13* in Arabidopsis transgenic plants leads to tolerance to drought stress [115]. Altogether, studies have indicated biological functions of the HD-ZIP I members consist of response to drought, salt, cold, and ABA [97].

To validate the rate of relative changes in the expression of HD-ZIP genes in response to drought stress, relative expression of six genes which showed significant differential expression in RNA-seq assay (Table S2) was examined using real-time qRT-PCR. This examination showed that four HD-ZIP genes were significantly upregulated and two HD-ZIP genes were significantly dowregulated in response to drought stress (Fig. 7B) similar to what was recorded in RNA-seq assay. The four upregulated genes showed 3.15 to 9.05 fold changes and the two dowreulated genes showed − 2.6 to −5.7 fold changes. Therefore, we can conclude that the six differntially expressed genes are drought-responsive HD-ZIP genes in safflower. The identification of these genes has a promising role in the future strategic breeding programs to improve more drought-tolerant cultivars of safflower. Our study again reveals that the HD-ZIP genes, particularly those in I and II subfamilies play a crucial role in plant response to abiotic stresses. In wheat, HD-ZIP I proteins (*TaHDZIPI-3*, *−4* &*−5*) bind to regulatory elements of responsive genes under drought stress [15]. Overexpression of the *ZmHDZ4* and *ZmHDZ10* genes in transgenic rice induced drought tolerance [116]. *NaHD20* gene belonging to the HD-ZIP I subfamily leads to upregulation of *NaNCED1*, *NaOSM1* and *NaLTP1* genes, as well as to ABA accumulation in tobacco (*Nicotiana attenuata*) leaves in response to drought stress [46]. Overexpression of the *OsHOX22* gene from the HD-ZIP I subfamily in transgenic rice lines leads to an increase in the content of ABA and a decrease in drought tolerance, and hence downregulation of the *OsHOX22* gene reduces the amount of ABA and enhances drought tolerance [117]. We couldn’t detect significant drought-responsiveness of any members of HD-ZIP III and IV groups in safflower RNA-seq assay. Seemingly, HD-ZIP III and IV members in comparison to HD-ZIP I and II members maybe play a lesser role in abiotic stresses. There are few reports on the definitive roles of these two HD-ZIP subfamilies in abiotic stress reponse. Overexpression of *AtEDT1/HDG11* belonging HD-ZIP IV in *Brassica oleracea* var. *alboglabra* elevated drought tolerance [118]. This gene indirectly regulates stomatal density and water-use efficiency [119], and via interaction with BIL9 improves drought tolerance [120].

## Conclusions

Since the HD-ZIP proteins and their expression profile weren’t analyzed in safflower, we studied these transcription factors both in silico and experimentally. We could identify 47 HD-ZIP proteins, from four classical subfamilies, encoded by safflower genome. Significant synteny patterns existed among HD-ZIPs of safflower and other plant species. Five pairs of duplicated genes were detected, most of which were emerged due to segmental (genome-wide) expansion. Most of safflower HD-ZIPs show interaction with each other or with other TFs and functional proteins that are involved in organogenesis and vital biological processes. Amongst members of different HD-ZIP subfamilies, only members of subfamily I (*n* = 7) and II (*n* = 1) responded to drought stress at seedling stage. These findings pave the way for functional analyses of safflower HD-ZIP genes and for application of their potential in regulating different biological processes, particularly abiotic stresses.

## Supplementary Information

Supplementary Material 1.

Supplementary Material 2

Supplementary Material 3

## Data Availability

Data is provided within the manuscript or supplementary information files or available from the corresponding author upon reasonable request. The RNAseq metadata are available in the NCBI repository under the accession number ID PRJNA1230082.

## Abbreviations

BLAST: Basic Local Alignment Search Tool
BP: Biological Process
CC: Cellular Component
DEG: Differentially Expressed Genes
HD: Homeodomain
HOX: Homeobox
LBD: Lab Binding Domain
LZ: Leucine Zipper
MF: Molecular Function
MSA: Muliple sequence alignment
MW: Molecular Weight
PPI: Protein-Protein Interaction
TF: Transcription Factor
