# Supplementary material for: Genome-wide identification and transcription profiling of safflower (Carthamus tinctorius L.) HD-ZIP gene family under water deficit
Source: BMC Genomics. 2025 Sep 29;26:874. doi: 10.1186/s12864-025-12060-4 (PMC12482594; doi:10.1186/s12864-025-12060-4)
Supplement: Supplementary file 1 — Supplementary Material 1. [file 12864_2025_12060_MOESM1_ESM.docx]

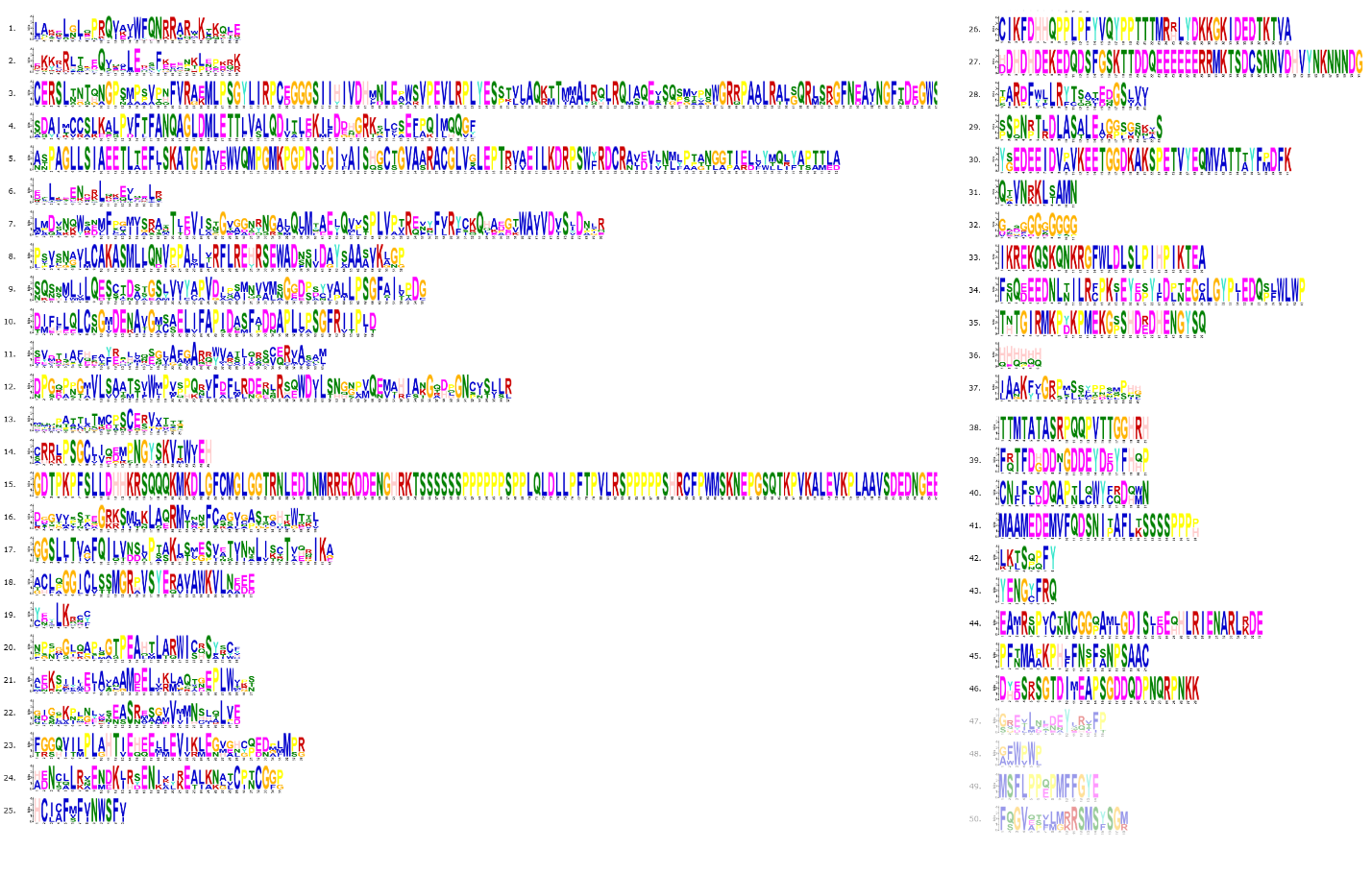


**Supplementary Fig. S1**. The logos of the conserved motifs of safflower HD-ZIP proteins.


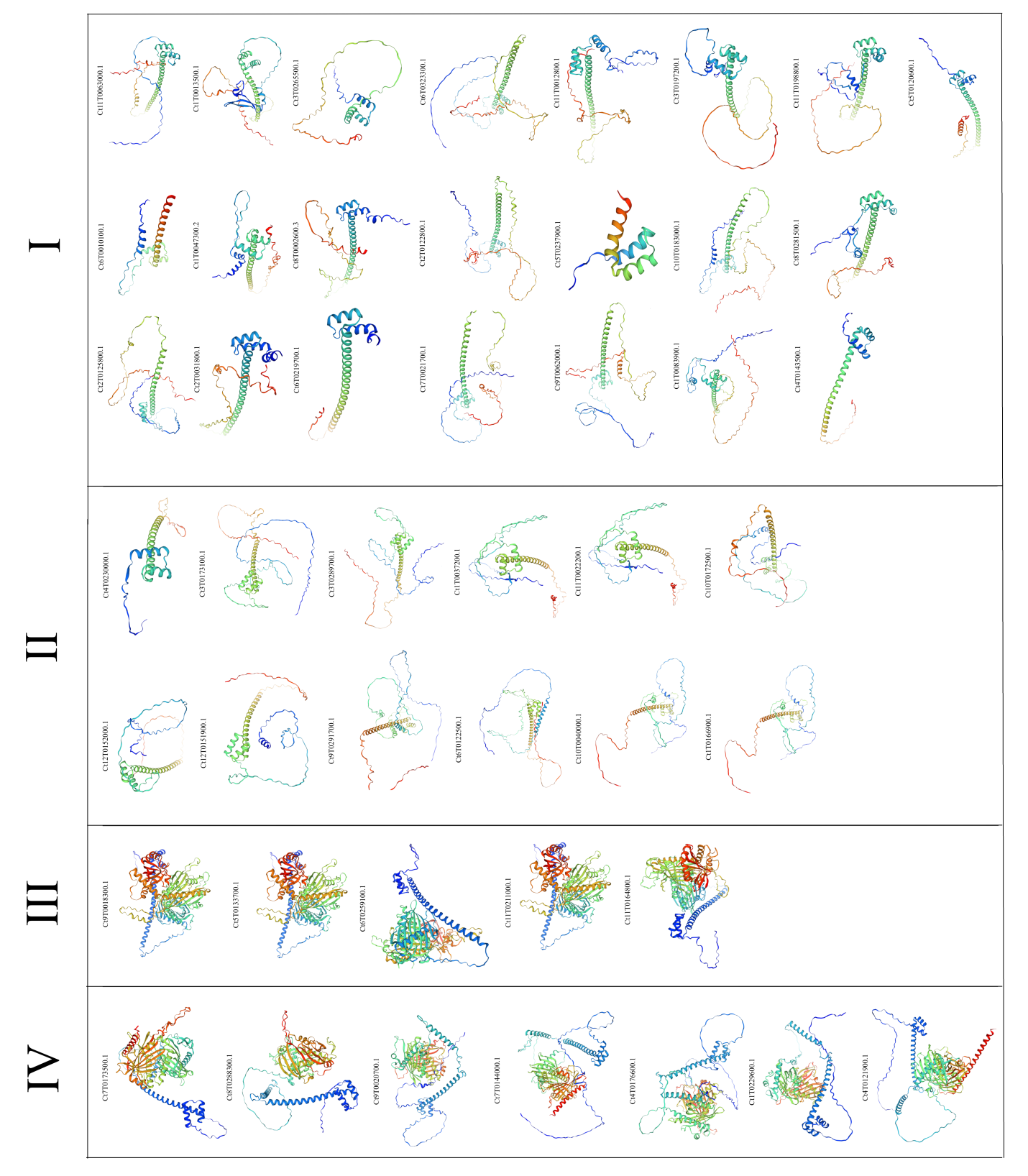


**Supplementary Figure S2**. Three-dimensional structure of HD-Zip proteins in safflower. Each box displays the HD-ZIP different family in safflower.

**
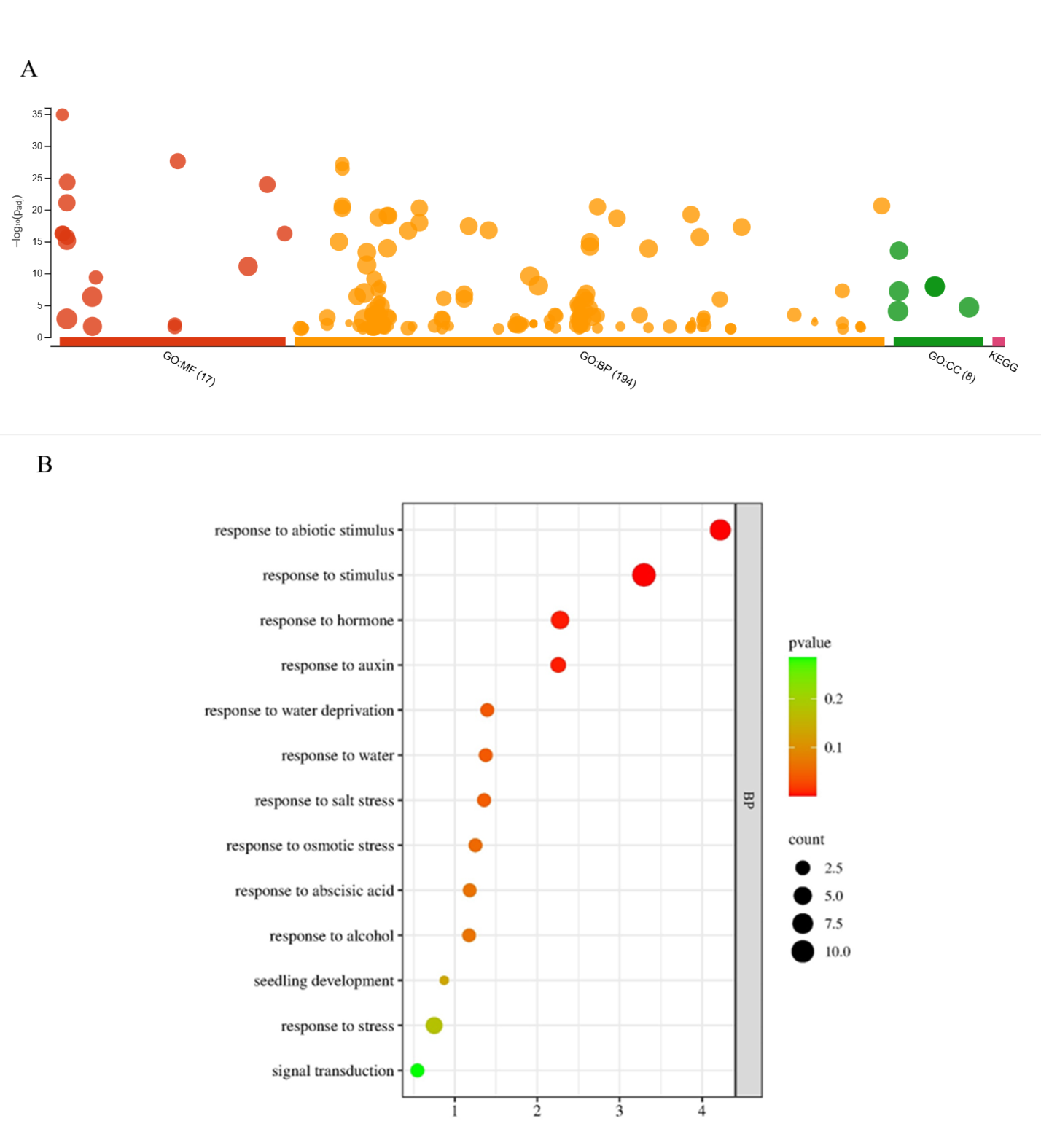
**

**Supplementary Fig. S3**. The ontology enrichments of 47 HD-ZIP genes in safflower. (A) Results of over representation analysis (ORA) of HD-ZIP genes in safflower presented by a Manhattan-like plot. The red color shows the molecular function (MF), the orange color shows the biological process (BP), and the green color shows the cellular component (CC). (B) Important biological processes mediated by HD-ZIP proteins in safflower.
