# Supplementary material for: Genome-wide identification and transcription profiling of safflower (Carthamus tinctorius L.) HD-ZIP gene family under water deficit
Source: BMC Genomics. 2025 Sep 29;26:874. doi: 10.1186/s12864-025-12060-4 (PMC12482594; doi:10.1186/s12864-025-12060-4)
Supplement: Supplementary file 2 — Supplementary Material 2 [file 12864_2025_12060_MOESM2_ESM.docx]

**Supplementary Table S1.** HD-ZIP subfamilies, subgroups, gene members of each subfamily, and their reported function in *A*. *thaliana*.

| Subfamily | Subgroups | Members of *A*. *thaliana* | Motif/Domain | Regulatory function | Reference |
| --- | --- | --- | --- | --- | --- |
| I | 7: α, β1, β2, γ, δ, ε, φ | 17: *AtHB1/HAT5*, *AtHB3/HAT7*, *AtHB5-AtHB7*, *AtHB12*, *AtHB13*, *AtHB16*, *AtHB20*- *AtHB23*, *AtHB40*, *AtHB51*-*AtHB54* | HD and LZ domains, WFQNRR motif | signal transduction, plant growth and development, plant tolerance to environmental stresses, α, β, γ subgroups are involved in response to drought stress | (19, 25, 26, 29, 30, 31, 32) |
| II | 5: α, β, γ, δ, ε | 9: *AtHB2/HAT4*, *ATHB4*, *HAT1-HAT3*, *HAT9*, *HAT14*, *HAT17, HAT22* | HD and LZ domains, CPSCE motif | plant response to light, shade and abiotic stresses, response to abiotic stresses such as drought, salinity, and plant hormones (ethylene, jasmonic acid, and ABA), response to drought stress | (17, 25, 33, 34, 35) |
| III | 3: α,β, γ | *5: AtHB8*, *PHAVOLUTA/AtHB9*, *PHABULOSA/AtHB14*, *CORONA/AtHB15,* *REVOLUTA/IFL1* | HD, START, SAD domains, MEKHLA motif | secondary cell wall biosynthesis | (36, 37, 38) |
| IV | 6: α, β, γ, ζ, ε, δ | *16: GL2/AtHB10*, *AtML1*, *ANL2*, *PDF2*, *HDG1*-*HDG5*, *HDG6/FWA,* *HDG7–HDG12* | HD, LZ, START, SAD domain | biotic stresses, transcriptional control of epidermal and sub-epidermal cell fate, anthocyanin accumulation, lipid transport, cuticle biosynthesis | (39, 40) |

**Supplementary Table S2.** Primer sequences of selected up-and down-regulated genes for validation of RNA-seq results by real-time qRT-PCR.

| Gene accessions | Sequence (5'→3') | Mean Tm (°C) | Amplicon size (bp) | Expression in RNAseq assay |
| --- | --- | --- | --- | --- |
| *CtHDZIP26* | CAAATGCTGAAAGAAAAGTTGG | 59.80 | 238 | UP |
|  | ATGAATCTTGATGTGGGCTGTT |  |  |  |
| *CtHDZIP7* | GATTGGGAGGAAACTACCTGTG | 58.90 | 151 | UP |
|  | TGTGTATGCTAACTACTGGGAAAA |  |  |  |
| *CtHDZIP37* | TTCCAAAACTGCCTTGATCTCT | 58.50 | 99 | UP |
|  | CAAAATTGACCTTGTTTATCTAATTC |  |  |  |
| *CtHDZIP3* | GGATGGAAGGATTGGAGAATG | 60.05 | 166 | UP |
|  | ACCAGAAAGCAAGGAGAAAAGA |  |  |  |
| *CtHDZIP41* | AGCCGTTAAATCACACAATCAA | 59.50 | 162 | DOWN |
|  | CGACAAGACAAATTAAGAAGAGAGAA |  |  |  |
| *CtHDZIP9* | AGACGCTTGAGAGGAACTTTG | 58.90 | 128 | DOWN |
|  | CCATCTTGCCCTTCTGTTTT |  |  |  |

**Supplementary Table S3.** The HD-ZIP genes classified in *C. tinctorius* based on protein homology to *A. thaliana.*

| Gene ID | CtHDZIP name | *A.thaliana* homolog | %identity | Protein id | Unigene | HD-ZIP subfamily |
| --- | --- | --- | --- | --- | --- | --- |
| *Ct11T0063000.1* | *CtHDZIP41* | *AT1G69780* | 45.12 | Q8LC03 | HB13 | I |
| *Ct6T0010100.1* | *CtHDZIP21* | *AT1G69780* | 63 | Q8LC03 | HB13 | I |
| *Ct2T0125800.1* | *CtHDZIP9* | *AT1G69780* | 60.94 | Q8LC03 | HB13 | I |
| *Ct1T0013500.1* | *CtHDZIP1* | *AT1G69780* | 46.62 | Q8LC03 | HB13 | I |
| *Ct1T0047300.2* | *CtHDZIP3* | *At4G36740.1* | 57.67 | O23208 | HB40 | I |
| *Ct2T0031800.1* | *CtHDZIP7* | *AT2G46680* | 58.25 | P46897 | HB-7 | I |
| *Ct3T0265500.1* | *CtHDZIP12* | *AT2G46680* | 61.17 | P46897 | HB-7 | I |
| *Ct8T0002600.3* | *CtHDZIP29* | *AT2G46680* | 60.95 | P46897 | HB-7 | I |
| *Ct6T0219700.1* | *CtHDZIP23* | *AT2G46680* | 44.02 | P46897 | HB-7 | I |
| *Ct6T0323300.1* | *CtHDZIP25* | *AT3G01470* | 43.16 | Q02283 | HAT5/HB-1 | I |
| *Ct2T0122800.1* | *CtHDZIP8* | *AT3G01470* | 41.63 | Q02283 | HAT5/HB-1 | I |
| *Ct7T0021700.1* | *CtHDZIP26* | *AT3G01470* | 71.72 | Q02283 | HAT5/HB-1 | I |
| *Ct11T0012800.1* | *CtHDZIP39* | *AT3G01470* | 49.13 | Q02283 | HAT5/HB-1 | I |
| *Ct5T0237900.1* | *CtHDZIP20* | *AT3G01470* | 75.56 | Q02283 | HAT5/HB-1 | I |
| *Ct9T0062000.1* | *CtHDZIP34* | *AT3G01470* | 39.19 | Q02283 | HAT5/HB-1 | I |
| *Ct3T0197200.1* | *CtHDZIP11* | *AT4G40060* | 62.67 | Q940J1 | HB16 | I |
| *Ct10T0183000.1* | *CtHDZIP38* | *AT4G40060* | 37.1 | Q940J1 | HB16 | I |
| *Ct1T0083900.1* | *CtHDZIP4* | *AT4G40060* | 48.31 | Q940J1 | HB16 | I |
| *Ct11T0198800.1* | *CtHDZIP43* | *AT5G03790* | 57.62 | Q9LZR0 | HB51 | I |
| *Ct8T0281500.1* | *CtHDZIP30* | *AT5G03790* | 52.31 | Q9LZR0 | HB51 | I |
| *Ct4T0143500.1* | *CtHDZIP15* | *AT5G53980* | 41.59 | Q9FN29 | HB52 | I |
| *Ct5T0120600.1* | *CtHDZIP18* | *AT5G53980* | 47.54 | Q9FN29 | HB52 | I |
| *Ct4T0230000.1* | *CtHDZIP17* | *AT2G01430* | 51.15 | Q8S9N6 | HB17 | II |
| *Ct3T0173100.1* | *CtHDZIP10* | *AT3G60390* | 68.69 | P46602 | HAT3 | II |
| *Ct3T0289700.1* | *CtHDZIP13* | *AT4G16780* | 64.16 | Q05466 | HAT4/HB-2 | II |
| *Ct1T0037200.1* | *CtHDZIP2* | *AT4G37790* | 53.95 | P46604 | HAT22 | II |
| *Ct11T0022200.1* | *CtHDZIP40* | *AT4G37790* | 50.46 | P46604 | HAT22 | II |
| *Ct10T0172500.1* | *CtHDZIP37* | *AT4G37790* | 62.5 | P46604 | HAT22 | II |
| *Ct12T0152000.1* | *CtHDZIP46* | *AT5G06710* | 59.32 | AAA56900.1 | HAT14 | II |
| *Ct12T0151900.1* | *CtHDZIP45* | *AT5G06710* | 46.61 | AAA56900.1 | HAT14 | II |
| *Ct9T0291700.1* | *CtHDZIP35* | *AT5G06710* | 47.58 | P46665 | HAT14 | II |
| *Ct6T0122500.1* | *CtHDZIP22* | *AT5G06710* | 45.67 | P46665 | HAT14 | II |
| *Ct10T0040000.1* | *CtHDZIP36* | *AT5G06710* | 49.32 | P46665 | HAT14 | II |
| *Ct1T0166900.1* | *CtHDZIP5* | *AT5G06710* | 49.32 | P46665 | HAT14 | II |
| *Ct9T0018300.1* | *CtHDZIP32* | *AT1G52150* | 83.81 | Q9ZU11 | HB-15 | III |
| *Ct5T0133700.1* | *CtHDZIP19* | *AT1G52150* | 72.75 | Q9ZU11 | HB-15 | III |
| *Ct6T0259100.1* | *CtHDZIP24* | *AT1G52150* | 79.31 | Q9ZU11 | HB-15 | III |
| *Ct11T0211000.1* | *CtHDZIP44* | *AT4G32880* | 70.69 | Q39123 | HB-8 | III |
| *Ct11T0164800.1* | *CtHDZIP42* | *AT5G60690* | 81.26 | Q9SE43 | REV | III |
| *Ct7T0173500.1* | *CtHDZIP28* | *AT1G73360* | 58.61 | Q9FX31 | HDG11 | IV |
| *Ct8T0288300.1* | *CtHDZIP31* | *AT1G73360* | 52.87 | Q9FX31 | HDG11 | IV |
| *Ct9T0020700.1* | *CtHDZIP33* | *AT1G79840* | 57.07 | P46607 | GL2 | IV |
| *Ct7T0144000.1* | *CtHDZIP27* | *AT4G00730* | 49.64 | Q0WV12 | ANL2 | IV |
| *Ct4T0176600.1* | *CtHDZIP16* | *AT4G00730* | 67.38 | Q0WV12 | ANL2 | IV |
| *Ct1T0229600.1* | *CtHDZIP6* | *AT4G21750* | 67.45 | Q8RWU4 | ATML1 | IV |
| *Ct4T0121900.1* | *CtHDZIP14* | *AT4G21750* | 78.07 | Q8RWU4 | ATML1 | IV |

**Supplementary Table S4.** Duplication events within safflower HD-ZIP genes.

| Gene pair 1 | Gene pair 2 | Identity (%) | Duplication type |
| --- | --- | --- | --- |
| *CtHDZIP24* | *CtHDZIP32* | 0.765 | Segmental |
| *CtHDZIP4* | *CtHDZIP11* | 0.403 | - |
| *CtHDZIP21* | *CtHDZIP33* | 0.194 | - |
| *CtHDZIP6* | *HDZIP14* | 0.643 | Segmental |
| *CtHDZIP28* | *CtHDZIP31* | 0.549 | - |
| *CtHDZIP16* | *CtHDZIP27* | 0.534 | - |
| *CtHDZIP3* | *CtHDZIP15* | 0.235 | - |
| *CtHDZIP45* | *CtHDZIP46* | 0.692 | Tandem |
| *CtHDZIP2* | *CtHDZIP40* | 0.405 | - |
| *CtHDZIP10* | *CtHDZIP13* | 0.487 | - |
| *CtHDZIP5* | *CtHDZIP36* | 0.422 | - |
| *CtHDZIP7* | *CtHDZIP12* | 0.497 | - |
| *CtHDZIP23* | *CtHDZIP29* | 0.363 | - |
| *CtHDZIP30* | *CtHDZIP43* | 0.589 | - |
| *CtHDZIP1* | *CtHDZIP41* | 0.626 | Segmental |
| *CtHDZIP25* | *CtHDZIP34* | 0.594 | - |
| *CtHDZIP20* | *CtHDZIP26* | 0.602 | Segmental |
